# Synthesis of Novel C/D Ring Modified Bile Acids

**DOI:** 10.3390/molecules27072364

**Published:** 2022-04-06

**Authors:** Roselis A. Landaeta Aponte, Andreas Luxenburger, Scott A. Cameron, Alex Weymouth-Wilson, Richard H. Furneaux, Lawrence D. Harris, Benjamin J. Compton

**Affiliations:** 1 Ferrier Research Institute, Victoria University of Wellington, Lower Hutt 5010, New Zealand; landaeta.aponte@gmail.com (R.A.L.A.); andreas.luxenburger@vuw.ac.nz (A.L.); scott.cameron@vuw.ac.nz (S.A.C.); richard.furneaux@vuw.ac.nz (R.H.F.); 2 ICE Group, New Zealand Pharmaceuticals Ltd., 68 Weld Street, RD2, Palmerston North 4472, New Zealand; alex.weymouth-wilson@dextrauk.com

**Keywords:** bile acid, zinc carbenoids, cyclopropane, rearrangement, drug discovery

## Abstract

Bile acid receptors have been identified as important targets for the development of new therapeutics to treat various metabolic and inflammatory diseases. The synthesis of new bile acid analogues can help elucidate structure–activity relationships and define compounds that activate these receptors selectively. Towards this, access to large quantities of a chenodeoxycholic acid derivative bearing a *C*-12 methyl and a *C*-13 to *C*-14 double bond provided an interesting scaffold to investigate the chemical manipulation of the C/D ring junction in bile acids. The reactivity of this alkene substrate with various zinc carbenoid species showed that those generated using the Furukawa methodology achieved selective α-cyclopropanation, whereas those generated using the Shi methodology reacted in an unexpected manner giving rise to a rearranged skeleton whereby the C ring has undergone contraction to form a novel spiro–furan ring system. Further derivatization of the cyclopropanated steroid included *O*-7 oxidation and epimerization to afford new bile acid derivatives for biological evaluation.

## 1. Introduction

Bile acids are amphiphilic compounds synthesized from cholesterol in the liver. These steroidal molecules possess both hydrophobic and hydrophilic regions enabling them to form micelles and function as physiological detergents for the absorption, distribution, metabolism and excretion of nutrients [1]. Bile acids also act as signaling molecules that activate the host cell receptors farnesoid X receptor (FXR) [2,3] and Takeda G protein-coupled receptor (GPCR) 5 [4,5] (TGR5; also known as G protein-coupled bile acid receptor 1, GPBAR1). FXR, a member of the nuclear receptor superfamily, is expressed at high levels in the liver and intestine and plays a central role in bile acid homeostasis alongside regulating various aspects of lipid [6,7] and glucose metabolism [7,8,9] as well as being involved in anti-inflammatory [10] and anti-fibrotic [11] activities. TGR5 belongs to the class A GPCR subfamily and is ubiquitously expressed in many tissues. Activation of TGR5 results in increased energy expenditure in adipose tissue and the secretion of glucagon-like peptide 1 (GLP-1), which is implicated in glucose metabolism and insulin sensitivity [12]. Consequently, dual FXR/TGR5 agonists were considered potential therapeutics for the treatment of metabolic disorders such as hypercholesterolemia, hypertriglyceridemia and type 2 diabetes. Concomitant activation of TGR5, however, presents with adverse effects such as pruritus [13], cholesterol gallstone formation [14] and gallbladder overfilling [15]. To avoid such side-effects, the development of new therapeutics to treat these diseases hinge on the ability to target these receptors selectively. Our approach towards achieving this was to synthesize a library of compounds with a modified C/D ring junction to be screened against these receptors to help elucidate key structure–activity relationships.

**Figure 1:**
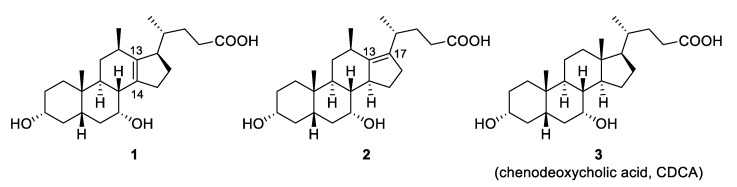
Isomeric, unsaturated chenodeoxycholic acid analogues **1** and **2** provide an interesting steroidal skeleton for chemical manipulation.

**Figure 2:**
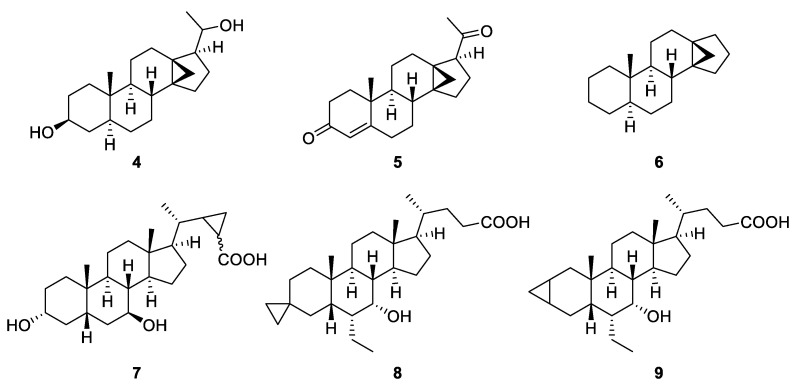
Examples of cyclopropane-containing androstanes and bile acids.

As part of a program to develop new bile acids with targeted activities, we established a scalable route to the two novel alkenes **1** and **2** (Figure 1) from cholic acid via a Nametkin-type rearrangement [16,17]. Of the various chemical manipulations afforded to olefins, we were particularly interested in applying those that result in the formation of new ring systems. Functionalizing the bile acids in this manner would not only afford a new class of compound to probe drug–receptor interactions but also advance investigations into the chemical reactivity of tetrasubstituted double bonds within the steroidal superstructure. Of the two chenodeoxycholic acid (CDCA, **3**) derivatives isolated, the Δ^13(14)^-scaffold **1** provided a unique opportunity to investigate the cycloaddition of methylene across the C/D ring junction to generate bile acid propellanes with altered lipophilicity and structural conformations from the parent compound. To the best of our knowledge, there are no published examples of bile acid propellanes. The only known androstane-derived propellanes are the β-facing cyclopropanes **4**–**6** (Figure 2), which were generated from deamination and a stereospecific rearrangement of 5α-H,18β-aminomethyl precursors [18,19,20]. Apart from a diastereomeric mixture of bile acids containing a side-chain cyclopropyl group across *C*-22 and *C*-23 (**7**) [21] and a spiro–cyclopropanyl derivative of obeticholic acid (**8**) [22], the only reported example of a bile acid containing a cyclopropyl at any position across the steroidal skeleton is compound **9** [22] (Figure 2).

Cyclopropanes, which can be formed with high diastereo- and enantiocontrol (as reviewed in [23,24]), have gained much attention in the fields of organic synthesis, medicinal chemistry and materials science for their interesting and unique properties [25]. The cyclopropanation of olefins can be catalyzed by various transition metal catalysts including zinc, copper, gold, palladium, and rhodium with others, such as ruthenium, iron, nickel, cobalt, titanium and yttrium, also having roles in cyclopropane formation [26]. Of these, zinc reagents often offer an inexpensive and less toxic approach to perform this functionalization. Founded on the seminal work by Emschwiller [27], who in 1929 showed diiodomethane and zinc react to form an iodomethylzinc species, and Simmons and Smith [28,29] (for a review of the Simmons−Smith cyclopropanation, see [30]) who later reported the formal cycloaddition of methylene across various olefins by treatment of diiodomethane with the zinc−copper couple (for a review on the uses of zinc carbenoids in stereoselective synthesis, see [31]); new organozinc carbenoids have been successfully employed to cyclopropanate traditionally unreactive alkenes, expanding the scope of suitable olefinic substrates. Among these are the methods developed by the groups of Furukawa [32,33], Wittig and Denmark [34], Shi [35,36] and Charette [37].

Beyond the reactivity of the carbenoid species, the olefin substrate can profoundly influence cyclopropane stereoselectivity and product yield. Although electron-rich tetrasubstituted olefins can be favored through electronic factors, steric constraints can hinder reaction yields. To overcome such limitations, directing groups (i.e., those which contain Lewis-basic heteroatoms) can be used to chelate metal carbenoids positioning them within proximity of the olefin in order to execute the ring-forming step. In structure **1**, we envisioned the homo-allylic alcohol at *O*-7 may provide such a handle.

## 2. Results and Discussion

**Scheme 1:**
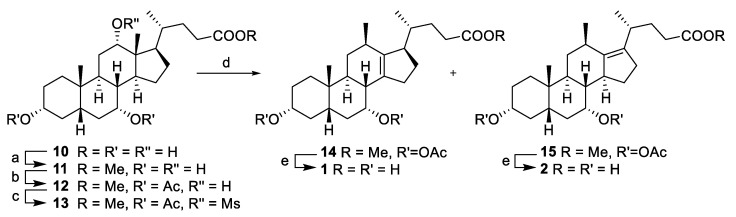
Nametkin-type rearrangement of cholic acid leads to two major isomeric alkenes: (**a**) MeOH, *p*-TsOH·H_2_O, 65 °C, 18 h; (**b**) Ac_2_O (2.2 equiv.), pyr, DMAP, 44%; (**c**) MsCl, pyr, 3 °C, 98%; (**d**) AcOH, NaOAc, 100 °C; (**e**) (i) NaOH, MeOH; (ii) HCl (aq), 0 °C, 7% (**1** from **12**) and 13% (**2** from **12**).

In accordance with published procedures [16], our synthetic efforts began with cholic acid (**10**), which was suitably protected to allow selective mesylation at *C*-12 to give **13** (Scheme 1). Exposure to a sodium acetate/acetic acid buffer at 100 °C afforded the isomeric Δ^13(14)^- and Δ^13(17)^ -alkenes **14** and **15** in a ratio of 1:2 as the major products. Hydrolysis of the methyl ester and acetate protecting groups followed by fractional crystallization from hot ethyl acetate/methanol mixtures afforded multi-gram quantities of **1** (7% from **13**) and **2** (13% from **13**) for further derivatization.

**Scheme 2:**
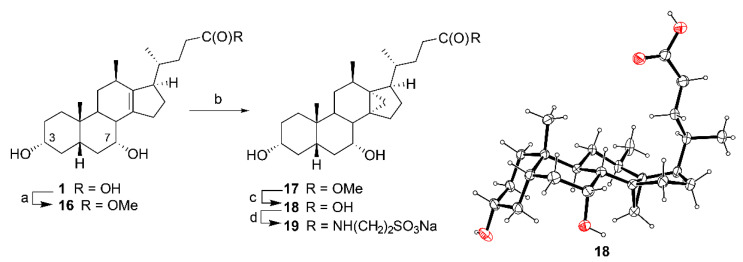
Stereoselective cyclopropanation and taurine conjugation of the rearranged cholic acid: (**a**) (i) Diazald, Et_2_O, KOH (aq), EtOH, 65 °C, (ii) EtOAc, (iii) AcOH, 80%; (**b**) Et_2_Zn, CH_2_I_2_, CH_2_Cl_2_, reflux, 4 h, 78%; (**c**) NaOH, MeOH/H_2_O, HCl, 92%; (**d**) (i) isobutyl chloroformate (1.2 equiv.), Et_3_N (4.4 equiv.), THF, −4 to −10 °C, 1 h; (ii) taurine (2.4 equiv.) in water, 18 h, 40%. ORTEP plot of the X-ray crystal structure of **18** (CCDC 2150664) shown with 30% probability ellipsoids.

With the rearranged precursor bile acids **1** and **2** in hand, we turned our attention towards cyclopropane formation with the aim of using Zn-carbenoids to effect this transformation. To avoid unwanted Zn-chelation via the carboxylic acid, **1** was first treated with diazomethane to afford the corresponding methyl ester **16** in excellent yield (Scheme 2). The first attempt to cyclopropanate alkene **16** employed the Furukawa methodology whereby the corresponding Zn-carbenoid was formed in situ by reaction of Et_2_Zn and diiodomethane. Employing toluene as the solvent (80 °C, 42 h) afforded cyclopropane **17** exclusively in 73% yield; however, performing the same reaction in refluxing dichloromethane gave a similar quantity of product after only 4 h (Scheme 2). Given that Furukawa carbenoids are renowned for their stereospecificity and high reactivity with electron-rich olefins, it was not surprising that a high yield of a single cyclopropane diastereomer was obtained from this reaction. Although the facial orientation of the cyclopropane moiety of **17** could not be established by NMR spectroscopy, it was anticipated that the homo-allylic 7-OH group would direct cyclopropanation via the α-face. Hydrolysis of ester **17** produced carboxylic acid **18**, and suitable crystals were obtained for X-ray diffraction, which unequivocally proved the stereochemistry of the newly installed cyclopropane to be *cis* to the 7-OH as anticipated. This is the first reported example of an α-facing cyclopropane across the C/D ring junction of any androstane-containing motif. Because bile acid conjugates, typically as taurine and glycine salts, are more readily transported in biological systems, converting **18** into its taurine analogue was considered prudent for biological evaluation. This was achieved by first forming the carbonic anhydride of **18** in situ before reaction with taurine to afford conjugate **19**.

The orientation of the 7-OH functionality in bile acids can have a pronounced effect on its biological activity. Compared to its 7α-epimer, CDCA, the 7β-hydroxy group of ursodeoxycholic acid (UDCA) renders this bile acid much more hydrophilic and therefore less toxic to gut bacteria [38]. However, CDCA has been shown to fully activate FXR, whereas UDCA had negligible activity on this receptor [39]. To investigate how the 7-OH functionality in **18** can influence potential receptor-agonist activities, a small library of bile acid propellanes was sought for biological testing. For this purpose, inversion of the *C*-7 hydroxyl group was attempted via oxidation and stereoselective reduction in the ketone moiety.

**Scheme 3:**
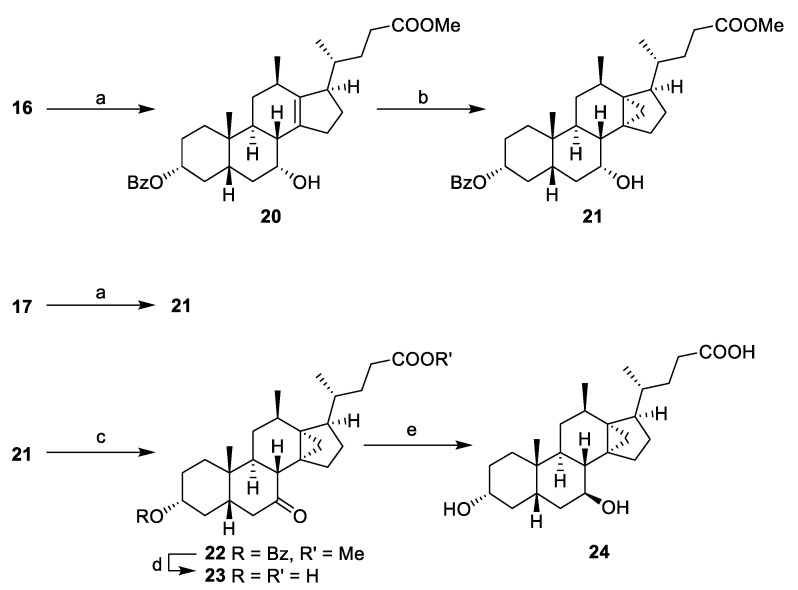
Synthesis of the 7-oxo and epimer analogues of the cholic acid-derived propellane: (**a**) BzCl, Pyr, toluene, 2 h; 93% (**16**→**20**); 94% (**17**→**21**); (**b**) Et_2_Zn, CH_2_I_2_, CH_2_Cl_2_, reflux, 4 h, 76%; (**c**) PCC (15 equiv.), CH_2_Cl_2_, 2 h, 95%; (**d**) NaOH, MeOH/H_2_O, then HCl, 75%; (**e**) Na(s), *i*-PrOH, 100 °C, 18 h, 21%.

A report [40] describing the selective oxidation of the 7-hydroxyl of CDCA, in the presence of an unprotected 3-hydroxyl group, with pyridinium chlorochromate (PCC) have attributed its regioselectivity to the fact that the oxidation of axial hydroxyl groups over equatorial hydroxyl groups is kinetically favored. Reaction times exceeding 15 min, however, result in formation of the di-keto product [40]. In our hands, applying the reported conditions to diol **18** resulted in formation of the 3,7-di-keto product while significant amounts of starting material remained in solution. To avoid formation of undesired oxidation products, the 3-OH was instead temporarily protected as a benzoyl ester (**16**→**20**) before subjection to the Furukawa carbenoid methodology, affording the cyclopropane intermediate **21** in 71% yield over the two steps (Scheme 3). Alternatively, intermediate **21** was furnished in a similar overall yield (73%) from direct selective mono-benzolyation of cyclopropane-diol **17**. With *O*-3 suitably protected and the cyclopropane installed, the oxidation of *O*-7 was screened using the common oxidants TEMPO/BAIB, NBS, KBr/NaOCl, PCC and Dess–Martin periodinane. From these, PCC proved to be the best oxidant for this substrate, affording the keto intermediate **22** in 95% yield, which was subsequently deprotected under basic reaction conditions to give **23**. Based on reports that elemental sodium can be used to selectively reduce keto moieties on steroids to give β-hydroxyl groups [41], **23** was treated with sodium in hot isopropanol. The desired 7β-OH analogue **24** was formed as the major product, albeit in a modest 21% yield. The stereochemistry of the 7-epimers (**18** and **24**) was determined by X-ray analysis (e.g., compound **18**) and by comparing the ^1^H NMR spectra of compounds **18** and **24**. For the 7α-OH-compound **18**, *H*-7_eq_ typically appears as a quartet due to its coupling with adjacent protons with similar coupling constants (δ 4.11 (q, *J* = 2.9 Hz, 1H, *H*-7_eq_)), whereas for **24**, *H*-7_ax_ couples to *H*-6_ax_ and *H*-8 at ca. 180° angles, and to *H*-6_eq_ at ca. 60°, appearing as a triplet of doublets (δ 3.63 (td, *J* = 11.6, 4.8 Hz, 1H, *H*-7_ax_)) and therefore distinguishable from its 7α-isomer.

**Scheme 4:**
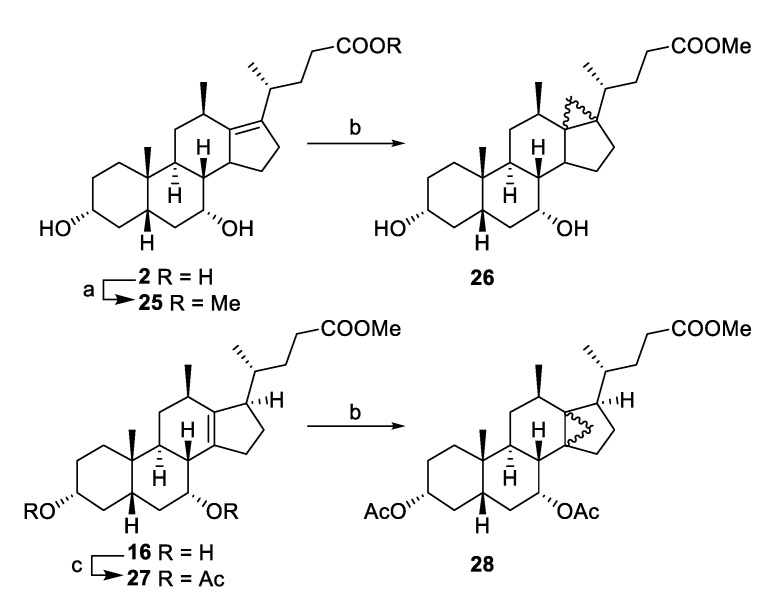
Stereoselective α-cyclopropantion of the Δ^13(14)^-bile acid occurs via 7-OH participation: (**a**) (i) Diazald, Et_2_O, KOH (aq), EtOH, 65 °C, (ii) EtOAc, (iii) AcOH, 93%; (**b**) Et_2_Zn, CH_2_I_2_, CH_2_Cl_2_, reflux, 5 h; (**c**) acetic anhydride, pyr, toluene, DMAP, 68%.

To investigate the importance of the proximity of the homo-allylic participating group at *O*-7 for cyclopropane formation, the Δ^13(17)^-alkene isomer **25**, with the alkene one carbon atom further removed from the directing group, was subjected to the Furukawa carbenoid for extended periods of time (Scheme 4). Monitoring the reaction by HPLC-MS showed only minor amounts (<10%) of putative cyclopropane formation as indicated by new peaks in the chromatogram with masses of M+14, with the bulk of the crude material comprised of starting material. The significance of the 7-hydroxyl group for directed carbene addition was further illustrated by subjecting the 3,7-di-OAc protected Δ^13(14)^-alkene isomer (**27**) (the protected form of **16**) to the same Furukawa conditions, but again, no significant amount of cyclopropane formation was observed. Notably, when protected alkene **27** was reacted with an excess of the Shi carbenoid, a reagent with higher reactivity than the Furukawa reagent towards isolated alkenes, no cyclopropane formation was detected.

**Scheme 5:**
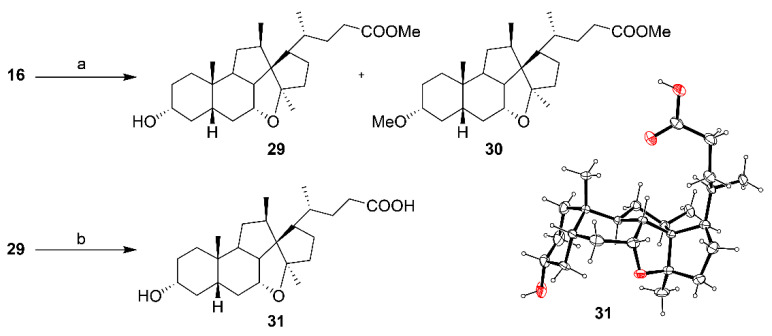
Unexpected formation of the spiro–furan ring system using the Shi carbenoid: (**a**) (i) Et_2_Zn, TFA, CH_2_Cl_2_, 10 min, 0 °C, (ii) CH_2_I_2_, 0 °C to r.t., 7% (for **29**) and 30% (for **30**; based on 90% purity); (**b**) NaOH, MeOH/H_2_O, HCl, 92%. ORTEP plot of the X-ray crystal structure of **31** (CCDC 2150665) shown with 30% probability ellipsoids.

In an attempt to improve directed cyclopropanation reactions of the Δ^13(14)^-substrate **16**, alternative zinc-carbenoids were explored. Reacting **16** with the Simmons–Smith carbenoid (IZnCH_2_I, 4 equivalents) in refluxing diethyl ether and monitoring the reaction by LCMS failed to generate any significant new products with only starting material being detected. Interestingly, employing the Shi carbenoid (CF_3_C(O)OZnCH_2_I, 10 equivalents) in refluxing dichloromethane resulted in the formation of a novel spiro-derivative (**29**) in 7% yield alongside its 3-OMe analogue **30** as the major product (30% yield, based on 90% purity) contaminated with a small amount of an unidentified impurity (Scheme 5) that was difficult to remove by normal-phase chromatography. Methylation of the 3-hydroxyl in **30** was not entirely unexpected as the alkylation of heteroatoms is a known side product when using excess reagent and/or prolonged reaction times owing to the high electrophilicity of the zinc carbenoid [42]. Hydrolysis of methyl ester **29** gave its corresponding acid (**31**) for which X-ray crystal data were obtained, confirming formation of the new steroidal skeleton as depicted in Scheme 5.

**Scheme 6:**
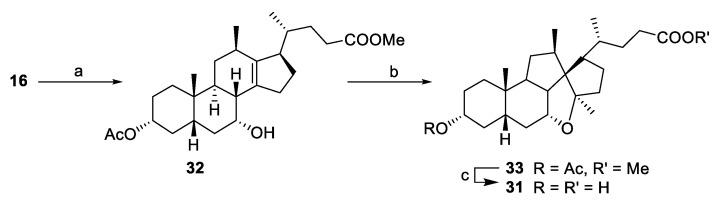
Improved route to spiro–furan ring system: (**a**) Acetic anhydride (18 equiv.), NaHCO_3_ (20 equiv.), THF, 40 °C, 33%; (**b**) (i) Et_2_Zn, TFA, CH_2_Cl_2_, 10 min, 0 °C, (ii) CH_2_I_2_, 0 °C to r.t., 2 h, 51% (based on purity of 90%); (**c**) NaOH, MeOH/H_2_O, HCl, 91%.

To improve the yield of **29**, it was proposed that protecting the 3-OH position of **16** would prevent formation of the methyl ether. Towards this, the selective protection of 3-OH was achieved with acetic anhydride and sodium bicarbonate in THF at 40 °C over 2 days. Increasing the reaction temperature to reflux gave a mixture of 7-OAc isomer and di-acetylated product after 2 h. With the 3-OAc compound (**32**) in hand, treatment with 10 equivalents of Shi’s Zn-carbenoid afforded the spiro derivative **33** (51%, based on 90% purity) contaminated with small amounts of unidentified impurities that were difficult to remove by chromatography on silica gel (Scheme 6). Deprotection afforded **31** in 83% yield.

**Scheme 7:**
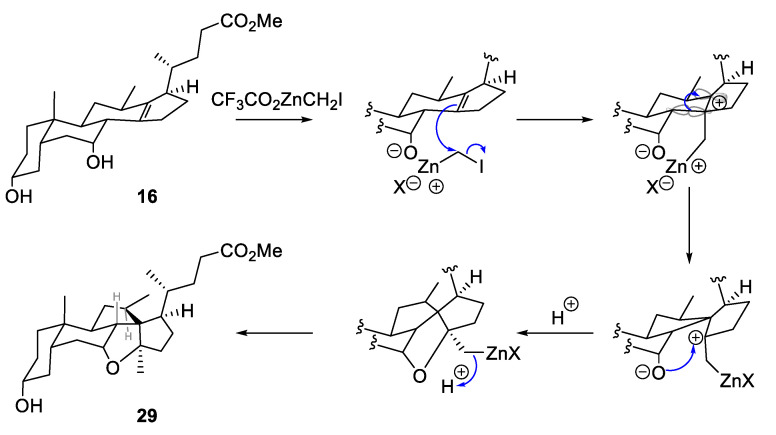
Proposed mechanism for rearrangement product from reacting the Shi Zn-carbenoid with the Δ^13(14)^-bile acid.

Because propellanes have been noted to rearrange under acidic conditions [43] it was postulated that **29** could have been formed by trifluoracetic acid-promoted rearrangement of a cyclopropane intermediate leading to the more thermodynamically stable product isolated. To evaluate this possibility, cyclopropane **17** was treated with either TFA or acetic acid and monitored by NMR spectroscopy (Supplementary Materials). However, after 5 min (TFA) or 4 h (AcOH), although the cyclopropane had been consumed, there was no evidence of **29** forming under these conditions. Instead, a potential mechanism leading to **29** is proposed to occur through coordination of the more electron deficient Shi carbenoid species to *O*-7, which then withdraws electron density from the (13,14)-double bond to form a carbocation at *C*-13 (Scheme 7). Concomitant migration of the (8,14)-σ-bond with the vacant *p*-orbital of the nascent carbocation at *C*-13, due to excellent orbital overlap, is followed by spiro-etherification of *O*-7 at the newly formed carbocation, then quenching of the resultant carbenoid species. This rearrangement process may be highly concerted based on the minimal atomic reorganization required, and to the best of our knowledge is an unprecedented and unexplored skeletal rearrangement in bile acid and steroid chemistry.

## 3. Conclusions

The stereoselective α-cyclopropanation of a Δ^13(14)^-bile acid alkene (**16** or its 3-OH-protected form **20**), in which the 7-OH is not protected, occurs readily when it is treated with the Furukawa carbenoid via assisted intramolecular addition. Applying the same conditions to either the isomeric Δ^13(17)^-bile acid **2** or to compound **27**, where the 7-OH is protected, abolishes cyclopropane formation showing the importance of the homo-allylic hydroxyl for directed methylene cycloaddition in this system. The cyclopropane product was further derivatized as the taurine conjugate and converted to a 7-keto-derivative and its 7β-epimer. In contrast to treatment with the Furukawa carbenoid, when the Δ^13(14)^-alkene (**16** or its 3-OH-protected form **32**) was exposed to the Shi carbenoid, the major product was an unexpected rearranged spiro–furan derivative, the structure of which was elucidated through X-ray crystallography. Overall, this study has resulted in bile acid analogues with altered C/D ring conformations, which are currently being investigated for their ability to selectively activate the bile acid receptors FXR and TGR5.

## 4. Materials and Methods

Proton (^1^H) and carbon (^13^C) NMR spectra were recorded on Bruker Avance (III)-500 MHz spectrometer. Chemical shifts are reported in ppm relative to Me_4_Si (TMS, δ 0.00 ppm), or residual solvent peaks as an internal standard set to δ 7.26 and 77.16 ppm (CDCl_3_), or δ 3.31 and 49.00 ppm (CD_3_OD), or δ 2.50 and 39.52 ppm (d_6_-DMSO) or δ 7.01 and 20.43 ppm (toluene-d_8_). Electrospray ionization (ESI) mass spectrometry (MS) experiments were performed on a Waters QTOF Premier mass spectrometer (Micromass, UK) under normal conditions. Sodium formate solution was used as calibrant for high resolution mass spectra (HRMS) measurements. Reactions were monitored by thin layer chromatography (TLC) using 0.2 mm silica gel (Merck Kieselgel 60 F254) precoated aluminum plates, using UV light, ammonium molybdate or potassium permanganate staining solution to visualize. Silver nitrate-impregnated TLC plates were prepared by dipping silica gel precoated aluminum plates in a 20% silver nitrate solution in acetonitrile and drying these in an oven at 120 °C. Silver nitrate-impregnated silica gel was prepared by dispersing silica gel in a solution of the corresponding amount of silver nitrate in acetonitrile and concentrating this mixture to dryness. Flash column chromatography was performed on Davisil^®^ silica gel (60, particle size 40–63 μm), or using Reveleris^®^ silica or C18 reversed phase flash cartridges on a Grace Reveleris^®^ automated flash system with continuous gradient facility. Solvents for reactions and chromatography were analytical grade and were used as supplied unless otherwise stated. Crystal structures were collected on an Agilent SuperNova diffractometer fitted with an EOS S2 detector, using CuKα radiation (1.54184 Å) at 120 K.

*Methyl 3α,7α-diacetoxy-12α-hydroxy-5β-cholan-24-oate* (**12**) [44]. To a solution of **11** [45] (535.3 g, 1.27 mol) in ethyl acetate (2.5 L) was added 4-(dimethylamino)pyridine (7.80 g, 63 mmol) and pyridine (290 mL, 3.55 mol). The mixture was cooled to 3 °C before the dropwise addition of acetic anhydride (270 mL, 2.78 mol) and the reaction mixture was warmed to r.t. (48 h). Water (1.8 L) was added and the organic layer was washed with water (1 L), 10% HCl (2 × 600 mL), brine (600 mL), dried (MgSO_4_) and concentrated. The product was purified via fractional precipitation from a mixture of ethyl acetate/petroleum ether (1:1.6) to afford the title compound **12** (283.4 g, 44%). ^1^H and ^13^C NMR spectra matched that previously reported [44].

*Methyl 3α,7α-diacetoxy-12α-[(methylsulfonyl)oxy]-5β-cholan-24-oate* (**13**) [46]. A solution of **12** (283.4 g, 559 mmol) in pyridine (1.7 L) was concentrated to approx. 1.4 L then cooled to 3 °C before the addition of methanesulfonyl chloride (94 mL, 1.19 mol). After stirring at r.t. (20 h) the reaction was quenched with water (100 mL) and ethyl acetate (600 mL), then concentrated. Water (1 L) and ethyl acetate (1 L) were added and the organic layer was washed with water (800 mL), 10% HCl (600 mL) and brine (800 mL). The organic layer was concentrated to dryness to afford the title compound **13** (319.8 g, 98%). ^1^H and ^13^C NMR spectra matched that previously reported [46].

*Methyl 3α,7α-diacetoxy-12β-methyl-18-nor-5β-chol-13(14)-en-24-oate* (**14**) *and methyl 3α,7β-diacetoxy-12β-methyl-18-nor-5β-chol-13(17)-en-24-oate* (**15**). To a suspension of mesylate **13** (319.8 g, 547 mmol) in acetic acid (320 mL) at 100 °C was added sodium acetate (136.8 g, 1.65 mol) and stirred (18 h) before concentrating from toluene (2 × 800 mL) then ethyl acetate (800 mL). Ethyl acetate (1 L) and water (600 mL) were added and the organic layer was washed with water (500 mL), brine (500 mL), dried (MgSO_4_) and concentrated to afford a crude sample containing the title compounds **14** and **15** as a ~1:2 mixture of isomers (respectively), which were used in the next step.

*3α,7α-Dihydroxy-12β-methyl-18-nor-5β-chol-13(17)-en-24-oic acid* (**1**) *and 3α,7α-dihydroxy-12β-methyl-18-nor-5β-chol-13(14)-en-24-oic acid* (**2**) [16]. To a solution of alkenes **14** and **15** in methanol (300 mL) was added NaOH (aq) (2M, 2.973 L, 5.95 mol) and the reaction mixture was stirred at 70 °C (18 h). The reaction was quenched with a solution of HCl (aq) (2M, 4.460 L, 8.92 mol) and the alkenes **1** (14.53 g, 7% from **13**) and **2** (27.85 g, 13% from **13**) were obtained separately by fractional precipitation from a mixture of methanol/ethyl acetate. ^1^H and ^13^C NMR spectra for each compound matched that previously reported [16].

*Methyl 3α,7α-dihydroxy-12β-methyl-18-nor-5β-chol-13(14)-en-24-oate* (**16**). An ethereal solution of diazomethane was prepared by distilling a solution of Diazald (1.00 g, 2.56 mmol) in a mixture of Et_2_O (15 mL) and 1.8M KOH (aq) (2:8 mL water/EtOH, 17.8 mmol). The freshly distilled diazomethane was added slowly to a suspension of alkene **1** (1.00 g, 2.5 mmol) in ethyl acetate (100 mL) at r.t. until complete conversion to its methyl ester. Excess diazomethane was quenched with acetic acid and the solution was concentrated from toluene (50 mL) followed by methanol (50 mL) to afford the title compound **16** (0.83 g, 80%). ^1^H NMR (500 MHz, CDCl_3_) δ 4.08 (q, *J* = 2.9 Hz, 1H), 3.63 (s, 3H), 3.43 (tt, *J* = 11.1, 4.4 Hz, 1H), 2.62–2.56 (broad signal, 1H), 2.37–2.05 (m, 9H), 2.03–1.84 (m, 4H), 1.83–1.72 (m, 2H), 1.72–1.60 (m, 4H), 1.52 (dt, *J* = 14.0, 2.2 Hz, 1H), 1.44–1.16 (m, 3H), 1.05–0.93 (overlapping signals: m, 2H and 1.03, d, *J* = 7.1 Hz, 3H), 0.92 (d, *J* = 6.8 Hz, 3H), 0.87 (s, 3H); ^13^C NMR (126 MHz, CDCl_3_) δ 174.5, 142.7, 137.9, 71.8, 68.1, 53.5, 51.4, 41.5, 41.0, 39.9, 35.6, 35.2, 34.9, 34.1, 33.6, 33.0, 32.7, 31.9, 31.3, 30.7, 25.2, 24.7, 22.3, 21.1, 18.9; HRMS(ESI) *m/z* calcd. for C_25_H_40_O_4_Na [M + Na]^+^: 427.2824, found 427.2825.

*Methyl 3α,7α-dihydroxy-12β-methyl-13α,14α-methylene-18-nor-5β-cholan-24-oate* (**17**). To Et_2_Zn (1M in hexanes, 2.5 mL, 0.25 mmol) in dichloromethane (10 mL) was added a solution of CH_2_I_2_ (0.23 mL, 2.8 mmol) in dichloromethane (5 mL), followed by addition of **16** (0.100 g, 0.247 mmol) in dichloromethane (5 mL) and the reaction mixture was stirred at 80 °C. After 4 h, the reaction was quenched with NH_4_Cl (20 mL), extracted with ethyl acetate (20 mL) and washed with brine (20 mL), dried (MgSO_4_) and concentrated. The residue was purified by flash chromatography on silica gel (ethyl acetate/petroleum ether, 0:10 to 8:2) to afford the title compound **17** (0.081 g, 78%). ^1^H NMR (500 MHz, CDCl_3_) δ 4.09 (q, *J* = 3.2 Hz, 1H), 3.64 (s, 3H), 3.46 (tt, *J* = 11.1 Hz, 4.5 Hz, 1H) 2.39 (ddd, *J* = 15.0, 10.4, 5.5 Hz, 1H), 2.24–2.10 (m, 4H), 1.99–1.92 (ddd, *J* = 14.8 Hz, 5.5 Hz, 3.9 Hz, 1H), 1.85–1.80 (m, 2H), 1.79–1.61 (m, 8H), 1.52–1.45 (m, 1H), 1.44–1.24 (m, 5H), 1.22 (d, *J* = 7.5 Hz, 3H), 1.20–1.08 (m, 2H), 1.01 (td, *J* = 14.3, 3.4 Hz, 1H), 0.87 (s, 3H), 0.85 (d, *J* = 6.6 Hz, 3H), 0.81–0.74 (m, 1H), 0.65 (d, *J* = 3.9 Hz, 1H), 0.35 (d, *J* = 3.9 Hz, 1H); ^13^C NMR (126 MHz, CDCl_3_) δ 174.2, 71.9, 69.0, 51.5, 50.5, 43.0, 41.0, 39.8, 39.5, 35.4, 34.8, 34.0, 33.7, 33.04, 32.99, 32.9, 32.6, 30.6, 30.1, 29.9, 27.1, 23.8, 22.8, 22.4, 20.9, 18.7; HRMS(ESI) *m/z* calcd. for C_26_H_43_O_4_ [M + H]^+^: 419.3161, found 419.3157.

*3α,7α-Dihydroxy-12β-methyl-13α,14α-methylene-18-nor-5β-cholan-24-oic Acid* (**18**). To a solution of **17** (0.045 g, 0.11 mmol) in MeOH (2 mL) was added NaOH (aq) (2M, 0.5 mL, 1 mmol). The reaction was stirred at 70 °C (2 h) before being quenched with a solution of HCl (aq) (2M, 1 mL, 2 mmol). The product was extracted with ethyl acetate (15 mL), washed with NaHCO_3_ (10 mL), brine (10 mL), dried (MgSO_4_) and concentrated to afford the title compound **18** (0.040 g, 92%). ^1^H NMR (500 MHz, CDCl_3_) δ 4.11 (q, *J* = 2.9 Hz, 1H), 3.48 (tt, *J* = 11.1, 4.4 Hz, 1H), 2.44 (ddd, *J* = 15.8, 10.5, 5.5 Hz, 1H), 2.27–2.13 (m, 4H), 2.00–1.93 (m, 1H), 1.86–1.62 (m, 9H), 1.52–1.45 (m, 1H), 1.45–1.25 (m, 5H), 1.23 (d, *J* = 7.5 Hz, 3H), 1.21–1.11 (m, 2H), 1.01 (td, *J* = 14.4, 3.6 Hz, 1H), 0.88–0.85 (overlapping signals: 0.86, d; 0.86, s, 6H), 0.84–0.76 (m, 1H), 0.66 (d, *J* = 4.1 Hz, 1H), 0.37 (d, *J* = 4.1 Hz, 1H); ^13^C NMR (126 MHz, CDCl_3_) δ 178.5, 72.0, 69.1, 50.5, 43.0, 41.1, 39.7, 39.5, 35.5, 34.8, 34.1, 33.8, 33.00, 32.95, 32.76, 32.67, 30.6, 30.1, 30.0, 26.9, 23.8, 22.8, 22.4, 20.9, 18.7; HRMS(ESI) *m/z* calcd for C_25_H_41_O_4_ [M + H]^+^: 405.3005, found 405.3005. Crystal data for C_25_H_40_O_4_ (M = 404.57 g/mol): orthorhombic, space group *P*2_1_2_1_2_1_, a = 11.4831(2) Å, b = 12.4753(2) Å, c = 19.9429(3) Å, V = 2856.92(8) Å^3^, Z = 4, T = 120(2) K, μ(CuKα) = 0.489 mm^−1^, 17,623 reflections measured (4.18° ≤ Θ ≤ 67.684°), 5577 unique (R_int_ = 0.0392), which were used in all calculations. The final R1 was 0.0434 (I > 2σ(I)) and wR2 was 0.1393 (all data).

*3α,7α-Dihydroxy-12β-methyl-13,14-methylene-18-nor-N-(2-sulfoethyl)-5β-cholan-24-amide* (**19**). Cyclopropane **18** (0.190 g, 0.470 mmol) was concentrated from triethylamine (0.16 mL, 1.15 mmol) then dissolved in THF (9 mL), cooled to −10 °C then isobutyl chloroformate (0.07 mL, 0.5 mmol) was added. After 1 h, triethylamine (0.16 mL, 1.15 mmol) was added followed by an aqueous solution of taurine (0.145 g, 1.14 mmol in 0.92 mL water) and the reaction was allowed to warm to r.t. (16 h). The mixture was concentrated, the residue was dissolved in MeOH (5 mL) and NaOH (2M, 1 mL, 2 mmol) was added. After 2 min, the mixture was concentrated then purified by flash chromatography on silica gel (chloroform/MeOH, 10:0 to 1:1) followed by chromatography on RP-C18 (water/MeOH, 20:1 to 1:4) to afford the title compound **19** (0.102 g, 40%). ^1^H NMR (500 MHz, CD_3_OD) δ 4.11 (q, *J* = 2.9 Hz, 1H), 3.60 (td, *J* = 6.9, 2.2 Hz, 2H), 3.45–3.37, (m, 1H), 2.98 (td, *J* = 7.1, 1.2, Hz, 2H), 2.36–2.27 (m, 1H), 2.27–2.19 (m, 3H), 2.12–2.00 (m, 2H), 1.94–1.86 (m, 2H), 1.85– 1.72 (m, 4H), 1.71–1.66 (m, 1H), 1.65–1.58 (m, 2H), 1.58–1.51 (m, 1H), 1.51–1.44 (m, 1H), 1.43–1.31 (m, 3H),1.29 (d, *J* = 7.5 Hz, 3H), 1.27–1.16 (m, 2H), 1.05 (td, *J* = 14.2, 3.4 Hz, 1H), 0.95 (s, 3H), 0.91 (d. *J* = 6.5 Hz), 0.89–0.81 (m, 1H), 0.70 (d, *J* = 4.3 Hz, 1H), 0.42 (d, *J* = 4.5 Hz, 1H); ^13^C NMR (126 MHz, MeOD) δ 176.3, 72.8, 70.1, 52.1, 51.6, 44.6, 42.8, 41.1, 40.5, 36.8, 36.7, 36.2, 36.1, 35.3, 35.1, 35.0, 34.3, 34.3, 31.8, 31.4, 31.3, 29.3, 25.7, 24.0, 23.4, 21.4, 19.2; HRMS(ESI) *m/z* calcd. for C_27_H_44_NNaO_6_S [M + Na]^+^: 533.2787, found 533.3123.

*Methyl 3α-benzoyl-7α-hydroxyl-12β-methyl-18-nor-5β-chol-13(14)-en-24-oate* (**20**). To a solution of **16** (1.00 g, 2.47 mmol) in a mixture of toluene (11 mL) and pyridine (12 mL) was added benzoyl chloride (0.44 mL, 3.8 mmol) and the mixture was stirred at r.t. (2 h). The reaction was quenched with water (20 mL), extracted with ethyl acetate (15 mL) and the organic layer was washed with NaOH (1M, 20 mL), water (20 mL) and brine (20 mL), dried (MgSO_4_), concentrated and the resulting residue was purified by flash chromatography on silica gel (ethyl acetate/ petroleum ether, 0:10 to 3:7) to afford **20** (1.172 g, 93%). ^1^H NMR (500 MHz, CDCl_3_) δ 8.02–7.97 (m, 2H), 7.52–7.47 (m, 1H), 7.42–7.35 (m, 2H), 4.85 (tt, *J* = 11.4, 4.6 Hz, 1H), 4.13 (q, *J* = 2.9 Hz, 1H), 3.64 (s, 3H), 2.66–2.60 (m, 1H), 2.59–2.48 (m, 1H), 2.43–2.32 (m, 3H), 2.32–2.23 (m, 2H), 2.19–2.10 (m, 2H), 2.05–1.97 (m, 2H), 1.96–1.88 (m, 1H), 1.88–1.76 (m, 4H), 1.75–1.66 (m, 2H), 1.66–1.59 (m, 1H), 1.58–1.52 (m, 2H), 1.32–1.21 (m, 2H), 1.17 (td, *J* = 14.4, 3.4 Hz, 1H), 1.09–0.96 (overlapping signals: m, 1H; 1.05, d, *J* = 6.8 Hz, 3H), 0.96–0.89 (overlapping signals: d, *J* = 6.8 Hz, 3H; 0.93, s, 3H); ^13^C NMR (126 MHz, CDCl_3_) δ 174.5, 166.1, 142.9, 137.9, 132.5, 130.9, 129.5, 128.1, 74.8, 68.1, 53.5, 51.4, 41.5, 40.7, 35.6, 35.4, 35.0, 34.9, 34.1, 33.6, 33.0, 32.7, 31.9, 31.4, 26.9, 25.2, 24.8, 22.3, 21.1, 18.9; HRMS(ESI) *m/z* calcd. for C_32_H_44_O_5_Na [M + Na]^+^: 531.3086, found 531.3090.

*Methyl 3α-benzoyl-7α-hydroxy-12β-methyl-13α,14α-methylene-18-nor-5β-cholan-24-oate* (**21**). From **17**: To a solution of **17** (0.418 g, 0.999 mmol) in toluene (5 mL) and pyridine (10 mL) was added benzoyl chloride (0.2 mL, 2 mmol) and the mixture was stirred at r.t. (1 h). Water (15 mL) and ethyl acetate (15 mL) were added and the organic layer was washed with NaOH (1M, 20 mL), water (20 mL) and brine (20 mL). The crude mixture was concentrated from toluene and MeOH, treated with Amberlyst^®^ A26 resin (hydroxide form) then purified by flash chromatography on silica gel (ethyl acetate/petroleum ether, 0:10 to 3:7) to afford compound **21** (0.493 g, 94%). From **20**: To Et_2_Zn (1M in hexanes, 2.5 mL, 2.5 mmol) in dichloromethane (15 mL) was added a solution of CH_2_I_2_ (0.57 mL, 6.9 mmol) in dichloromethane (10 mL), followed by the dropwise addition of **20** (0.590 g, 1.16 mmol) in dichloromethane (15 mL) and the reaction mixture was stirred at 80 °C (18 h). The reaction was quenched with NH_4_Cl (100 mL), extracted with ethyl acetate (2 × 50 mL) and the combined organic layers were washed with NaHCO_3_ (50 mL), brine (100 mL), dried (MgSO_4_) and concentrated. The residue was purified by flash chromatography on silica gel (ethyl acetate/petroleum ether, 0:10 to 3:7) to afford the title compound **21** (0.462 g, 76%). ^1^H NMR (500 MHz, CDCl_3_) δ 8.02 (d, *J* = 7.3 Hz, 2H), 7.50 (t, *J* = 7.3 Hz, 1H), 7.39 (t, *J* = 7.7 Hz, 2H), 4.83 (tt, *J* = 11.5, 4.4 Hz, 1H), 4.11 (broad signal, 1H), 3.65 (s, 3H), 2.49–2.33 (m, 2H), 2.27–2.21 (m, 1H), 2.20–2.04 (m, 3H), 2.04–1.95 (m, 1H), 1.95–1.71 (m, 8H), 1.70–1.62 (m, 1H), 1.60–1.45 (m, 3H), 1.45–1.31 (m, 2H), 1.23 (d, *J* = 7.4 Hz, 3H), 1.21–1.09 (m, 3H), 0.92 (s, 3H), 0.89–0.77 (overlapping signals: m, 1H; 0.85, d, *J* = 6.5 Hz, 3H), 0.69–0.64 (m, 1H), 0.45–0.38 (m, 1H); ^13^C NMR (126 MHz, CDCl_3_) δ 174.2, 166.1, 132.5, 131.0, 129.5, 128.1, 75.0, 69.0, 51.4, 50.4, 43.0, 40.9, 39.5, 35.3, 35.2, 34.9, 34.1, 33.7, 33.0, 32.9, 32.8, 32.6, 30.1, 29.9, 27.1, 27.0, 23.8, 22.8, 22.4, 20.8, 18.7; HRMS(ESI) m/z calcd. for C_33_H_46_O_5_Na [M + Na]^+^: 545.3243, found 545.3248.

*Methyl 3α-benzoyl-7-oxo-12β-methyl-13α,14α-methylene-18-nor-5β-cholan-24-oate* (**22**). Pyridinium chlorochromate (PCC) method: To a solution of **21** (0.190 g, 0.36 mmol) in dichloromethane (5 mL) was added silica gel (0.300 g) then PCC (1.200 g, 5.6 mmol) and the mixture was stirred at r.t. (2 h). The crude mixture was filtered, washed with ethyl acetate (30 mL) and the filtrate was washed with water and brine until the aqueous washing were clear affording the title compound **22** (0.181 g, 95%). Dess–Martin method: To a solution of **21** (0.045 g, 0.086 mmol) in dichloromethane (5 mL) was added Dess–Martin periodinane (1.4 equiv.) and the reaction was stirred at r.t. (4 h). The mixture was quenched with isopropanol (0.5 mL), concentrated and the residue was purified by flash chromatography on silica gel (ethyl acetate/toluene, 0:10 to 3:7) to afford the title compound **22** (0.029 g, 65%). ^1^H NMR (500 MHz, CDCl_3_) δ 8.04–7.95 (m, 2H), 7.56–7.48 (m, 1H), 7.45–7.36 (m, 2H), 4.94 (tt, *J* = 11.3, 4.7 Hz, 1H), 3.67 (s, 3H), 2.93–2.86 (m, 1H), 2.66 (d, *J* = 12.3 Hz, 1H), 2.41 (ddd, *J* = 15.4, 10.2, 5.4 Hz, 1H), 2.23–2.08 (m, 3H), 2.07–1.97 (m, 3H), 1.97–1.91 (m, 1H), 1.90–1.83 (m, 3H), 1.83–1.75 (m, 1H), 1.75–1.66 (m, 1H), 1.53–1.40 (m, 3H), 1.36–1.26 (m, 3H), 1.25–1.22 (overlapping signals: m, 1H; d, *J* = 7.5 Hz, 3H), 1.21 (s, 3H), 1.17–1.09 (m, 1H), 1.09–1.00 (m, 1H), 0.87–0.81 (overlapping signals: m, 1H; 0.84, d, *J* = 6.5 Hz, 3H), 0.25–0.20 (m, 1H); ^13^C NMR (126 MHz, CDCl_3_) δ 210.5, 174.2, 166.0, 132.8, 130.5, 129.5, 128.2, 73.6, 52.3, 51.5, 50.2, 45.1, 44.9, 39.4, 39.0, 34.8, 34.1, 33.8, 33.7, 32.9, 31.0, 30.9, 27.1, 26.4, 24.5, 23.1, 22.4, 20.6, 18.7; HRMS(ESI) m/z calcd. for C_33_H_45_O_5_ [M + H]^+^: 521.3267, found 521.3271.

*3α-Hydroxy-7-oxo-12β-methyl-13α,14α-methylene-18-nor-5β-cholan-24-oic acid* (**23**). To a solution of **22** (0.043 g, 0.083 mmol) in MeOH (2 mL) was added NaOH (aq) (2M, 0.5 mL, 1 mmol). The reaction was stirred at 70 °C (2 h) before being quenched with a solution of HCl (aq) (2M, 1 mL, 2 mmol). The product was extracted with ethyl acetate (15 mL), washed with NaHCO_3_ (15 mL), brine (15 mL), dried (MgSO_4_), and purified via flash chromatography on silica gel (acetone/dichloromethane + 1% AcOH, 0:10 to 1:1) to afford the title compound **23** (0.025 g, 75%). ^1^H NMR (500 MHz, CD_3_OD) δ 3.62–3.50 (m, 1H), 3.02 (dd, *J* = 1H), 2.79 (d, *J* = 12.3 Hz, 1H), 2.41 (ddd, *J* = 15.3, 10.1, 5.5 Hz, 1H), 2.30–2.10 (m, 3H), 1.99–1.86 (m, 5H), 1.86–1.75 (m, 2H), 1.72–1.62 (m, 2H), 1.56–1.48 (m, 1H), 1.39–1.09 (overlapping signals: m, 8H; 1.31, d, *J* = 7.5 Hz, 3H; 1.21, s, 3H), 0.90 (d, *J* = 6.6 Hz, 3H), 0.81 (d, *J* = 4.9 Hz, 1H), 0.22 (d, *J* = 4.6 Hz, 1H); ^13^C NMR (126 MHz, CDCl_3_) δ 214.3, 177.9, 71.5, 53.6, 51.7, 46.8, 46.1, 41.0, 40.5, 38.6, 35.8, 35.5, 35.0, 35.0, 34.1, 32.5, 32.3, 30.8, 28.7, 25.4, 24.1, 23.0, 21.0, 19.0; HRMS(ESI) *m/z* calcd. for C_25_H_37_O_4_^−^ [M − H]^−^: 401.2692, found 401.2713.

*3α,7β-dihydroxy-12β-methyl-13α,14α-methylene-18-nor-5β-cholan-24-oic Acid* (**24**). To a solution of **23** (0.022 g, 0.056 mmol) in isopropanol (5 mL) was added an excess of Na(s) (added until complete dissolution) and the reaction was heated to 100 °C (5 h). Once cooled, HCl (2M) was added until the pH = 1 and the product was extracted with ethyl acetate (3 × 15 mL). The organic layers were combined, washed with brine (10 mL), dried (MgSO_4_) and purified by flash chromatography on silica gel (acetone/dichloromethane + 1% acetic acid, 0:10 to 1:4) to afford the title compound **24** (5.0 mg, 21%). ^1^H NMR (500 MHz, CD_3_OD) δ 3.63 (td, *J* = 11.6, 4.8 Hz, 1H), 3.52 (tt, *J* = 10.5, 4.8 Hz, 1H), 2.39 (ddd, *J* = 15.1, 10.2, 5.4 Hz, 1H), 2.29–2.22 (m, 1H), 2.18–2.10 (m, 3H), 1.98–1.76 (m, 5H), 1.70–1.42 (m, 9H), 1.33–1.16 (m, 9H), 1.13–1.04 (m, 2H), 0.97 (s, 3H), 0.91 (d, *J* = 6.7 Hz, 3H), 0.63 (d, *J* = 4.4 Hz, 1H), 0.22 (dd, *J* = 4.5, 1.6 Hz, 1H). ^13^C NMR (126 MHz, CD_3_OD) δ 177.9, 72.4, 72.2, 51.3, 47.8, 43.8, 41.7, 38.6, 38.4, 37.3, 36.49, 36.46, 35.6, 35.5, 35.2, 34.3, 34.0, 31.1, 29.6, 28.8, 24.3, 24.3, 23.6, 21.1, 19.1; HRMS(ESI) *m/z* calcd. for C_25_H_39_O_4_ [M + H]^+^: 403.2848, found 403.2837.

*Methyl 3α,7α-dihydroxy-12β-methyl-18-nor-5β-chol-13(17)-en-24-oate* (**25**). An ethereal solution of diazomethane was prepared by distilling a solution of Diazald (1.00 g, 2.56 mmol) in a mixture of Et_2_O (15 mL) and 1.8M KOH (aq) (2:8 mL water/EtOH, 17.8 mmol). The freshly distilled diazomethane was slowly added to a suspension of **2** (0.459 g, 1.18 mmol) in ethyl acetate (100 mL) at r.t. until completion. Excess diazomethane was quenched with acetic acid and the solution was concentrated from toluene (50 mL) followed by methanol (50 mL) before concentrating to dryness. The product was purified by flash chromatography on silica gel (ethyl acetate/petroleum ether, 3:7 to 7:3) to afford the title compound **25** (0.446 g, 93%). ^1^H NMR (500 MHz, CDCl_3_) δ 3.92–3.85 (b.s., 1H), 3.60 (s, 3H), 3.40 (tt, *J* = 11.1, 4.4 Hz, 1H), 2.95 (h, *J* = 6.9 Hz, 1H), 2.66–2.23 (m, 3H), 2.23–2.08 (m, 6H), 2.00–1.93 (m, 1H), 1.93–1.85 (m, 2H), 1.84–1.77 (m, 1H), 1.70–1.61 (m, 2H), 1.61–1.54 (m, 3H), 1.54–1.48 (m, 1H), 1.39–1.27 (m 2H), 1.27–1.20 (m, 1H), 1.19 (d, *J* = 7.0 Hz, 3H), 1.05 (td, *J* = 11.2 Hz, 3.1 Hz, 1H), 0.97–0.89 (overlapping signals: m, 1H and 0.93, d, *J* = 6.7 Hz, 3H), 0.82–0.73 (overlapping signals: m, 1H and 0.76, s, 3H); ^13^C NMR (126 MHz, CDCl_3_) δ 174.3, 139.9, 134.2, 71.8, 67.0, 51.3, 49.3, 48.8, 41.2, 39.5, 36.3, 35.6, 35.1, 34.8, 34.4, 32.9, 32.5, 31.0, 30.6, 30.3, 30.0, 25.9, 22.8, 20.7, 19.6; HRMS(ESI) *m/z* calcd. for C_25_H_40_O_4_Na [M + Na]^+^: 427.2824, found 427.2824.

*Methyl 3α,7α-diacetyloxy-12β-methyl-18-nor-5β-chol-13(14)-en-24-oate* (**27**). To a solution of **16** (83 mg, 0.21 mmol) in toluene (4.5 mL) was added pyridine (1 mL) followed by 4-(dimethylamino)pyridine (8 mg, 0.065 mmol) then acetic anhydride (0.2 mL) at r.t. After 3 h, the reaction mixture was concentrated, dissolved in ethyl acetate (20 mL) then washed with 2M HCl (10 mL). The organic layer was washed with water, sat. NaHCO_3_ (20 mL), brine (20 mL), dried (MgSO_4_) and concentrated. The crude residue was purified by flash chromatography on silica gel (ethyl acetate/petroleum ether, 0:10 to 3:7) to afford the title compound **27** (68 mg, 68% yield). ^1^H NMR (500 MHz, CDCl_3_) δ 5.06 (q, *J* = 3.1 Hz, 1H), 4.63–4.54 (m, 1H), 3.63 (s, 3H), 2.55 (d, *J* = 9.1 Hz, 1H), 2.40 (d, *J* = 11.7 Hz, 1H), 2.32 (ddd, *J* = 15.0, 9.6, 5.3 Hz, 2H), 2.22–2.09 (m, 2H), 2.08–1.93 (overlapping signals: m, 4H and 1.99, s, 3H and 1.99, s, 3H), 1.92–1.81 (m, 2H), 1.78–1.60 (m, 6H), 1.60–1.54 (m, 1H), 1.53–1.40 (m, 2H), 1.26–1.17 (m, 1H), 1.11 (td, *J* = 14.4, 3.5 Hz, 1H), 1.02 (d, *J* = 6.8 Hz, 3H), 1.01–0.84 (overlapping signals: m, 1H and 0.91, d, *J* = 6.8 Hz, 3H and 0.90, s, 3H); ^13^C NMR (126 MHz, CDCl_3_) δ 174.3, 170.42, 170.39, 140.8, 137.6, 74.0, 70.8, 53.3, 51.3, 40.4, 39.3, 35.5, 34.6, 34.5, 33.4, 33.2, 32.53, 32.48, 31.5, 31.0, 26.8, 25.2, 25.0, 22.2, 21.4, 21.3, 21.0, 18.7; HRMS(ESI) *m/z* calcd. for C_29_H_44_O_6_Na [M + Na]^+^: 511.3036, found 511.3039.

*Methyl (R)-4-((1S,3aR,4aR,4a1R,5aS,7R,9aS,9bS,11R,11aR)-7-hydroxy-3a,9a,11-trimethylhexadecahydrobenzo[4,5]indeno[7,1-bc]cyclopenta[d]furan-1-yl)pentanoate* (**29**) *and methyl (R)-4-((1S,3aR,4aR,4a1R,5aS,7R,9aS,9bS,11R,11aR)-7-methoxy-3a,9a,11-trimethylhexadecahydrobenzo[4,5]indeno[7,1-bc]cyclopenta[d]furan-1-yl)pentanoate* (**30**). To a three-neck flask under argon and cooled to 0 °C was added dichloromethane (5 mL) then diethylzinc (1 M in hexanes, 10 mL) followed trifluoroacetic acid (0.4 mL in 10 mL of dichloromethane) and the mixture was left to stir for 10 min. A solution of diiodomethane (0.8 mL, 10 mmol) in dichloromethane (5 mL) was then added dropwise at 0 °C and the mixture stirred for 20 min before the addition of **16** (0.431 g, 1.07 mmol) in dichloromethane (5 mL) and the reaction was allowed to warm to r.t. After 4 h, the reaction was quenched with saturated NH_4_Cl (40 mL) and the aqueous layer was washed with ethyl acetate (2 × 30 mL). The combined organic layers were washed with NaHCO_3_ (40 mL), water (40 mL), brine (40 mL), dried (MgSO_4_) and concentrated. The crude residue was purified by flash chromatography on silica gel (ethyl acetate/petroleum ether, 1:4 to 4:1) to afford **29** (0.031 g, 7%) and **30** (0.103 g, 30% yield, based on a purity of 90%) as separate compounds. Compound **29**: ^1^H NMR (500 MHz, CDCl_3_) δ 4.21–4.13 (m, 1H), 3.65 (s, 3H), 3.55 (tt, *J* = 9.6, 4.9 Hz, 1H), 2.41 (ddd, *J* = 14.9, 9.4, 5,2 Hz, 1H), 2.32–2.19 (m, 3H), 2.01–1.97 (m, 1H), 1,91–1.85 (m, 1H), 1.85–1.81 (m, 1H), 1.81–1.76 (m, 3H), 1.76–1.70 (m, 1H), 1.70–1.64 (m, 4H), 1.59–1.50 (m, 2H), 1.49–1.43 (m, 1H), 1.43–1.39 (m, 1H), 1.39–1.33 (m, 2H), 1.33–1.26 (m, 1H), 1.25 (s, 3H), 1.22–1.15 (m, 1H), 1.14–1.04 (m, 1H), 1.00 (d, *J* = 7.3 Hz, 3H), 0.97 (d, *J* = 6.9 Hz, 3H), 0.96–0.89 (m, 1H), 0.89–0.78 (overlapping signals: m, 1H and 0.84, s, 3H); ^13^C NMR (126 MHz, CDCl_3_) δ 174.1, 94.4, 77.1, 70.4, 64.3, 51.49, 51.45, 50.7, 42.1, 39.7, 39.0, 38.6, 38.1, 35.8, 35.2, 33.6, 33.3, 32.7, 32.3, 30.2, 29.8, 27.5, 25.6, 20.4, 20.2, 16.9; HRMS(ESI) *m/z* calcd. for C_26_H_43_O_4_ [M+H]^+^: 419.3161, found 419.3155. Compound **30**: ^1^H NMR (500 MHz, CDCl_3_) δ 4.15 (dt, *J* = 5.2, 3.6 Hz, 1H), 3.64 (s, 3H), 3.26 (s, 3H), 3.13–3.05 (m, 1H), 2.40 (ddd, *J* = 14.9, 9.4, 5.2 Hz, 1H), 2.29–2.18 (m, 3H), 1.98–1.93 (m, 1H), 1.87–1.79 (m, 2H), 1.77–1.72 (m, 4H), 1.70–1.63 (m, 3H), 1.56–1.48 (m, 2H), 1.43–1.35 (m, 2H), 1.32–1.23 (m, 3H), 1.21 (s, 3H), 1.19–1.07 (m, 1H), 0.97 (ap. t, *J* = 7.0, 6H), 0.93–0.76 (overlapping signals: m, 2H and 0.83 (s, 3H); ^13^C NMR (126 MHz, CDCl_3_) δ 174.0, 94.3, 78.5, 77.2, 64.0, 55.4, 51.4, 51.3, 50.7, 41.9, 39.8, 39.4, 38.3, 35.8, 34.7, 34.0, 33.5, 33.4, 32.8, 32.7, 29.8, 27.4, 26.8, 25.5, 20.4, 20.2, 16.8; HRMS(ESI) *m/z* calcd. for C_27_H_44_O_4_Na [M + Na]^+^: 455.3137, found 455.3134.

*(R)-4-((1S,3aR,4aR,4a1R,5aS,7R,9aS,9bS,11R,11aR)-7-Hydroxy-3a,9a,11-trimethylhexadecahydrobenzo[4,5]indeno[7,1-bc]cyclopenta[d]furan-1-yl)pentanoic acid* (**31**). From **29**: To a solution of **29** (0.019 g, 0.045 mmol) in MeOH (2 mL) was added NaOH (aq) (2M, 0.5 mL, 1 mmol). The reaction was stirred at 70 °C (2 h) before being quenched with a solution of HCl (aq) (2M, 1 mL, 2 mmol). The product was extracted with ethyl acetate (10 mL), washed with water (10 mL) and brine (10 mL), dried (MgSO_4_) filtered and concentrated to afford compound **31** (0.017 g, 92%). From **33**: To a solution of **33** (0.030 g, 0.059 mmol based on purity of 90%,) in MeOH (2 mL) was added NaOH (aq) (2M, 1.5 mL, 3 mmol). The reaction was stirred at 70 °C (2 h) before being quenched with a solution of HCl (aq) (2M, 1 mL, 2 mmol). The product was extracted with ethyl acetate (10 mL), washed with water (10 mL) and brine (10 mL), dried (MgSO_4_) and concentrated to dryness. The residue was recrystallized from ethyl acetate/petroleum ether to afford compound **31** (0.022 g, 91%). ^1^H NMR (500 MHz, CD_3_OD) δ 4.27–4.23 (m, 1H), 3.50 (tt, *J* = 10.0, 4.2 Hz, 1H), 2.49–2.36 (m, 2H), 2.36–2.22 (m, 2H), 1.99–1.86 (m, 7H), 1.75–1.60 (m, 6H), 1.55–1.47 (m, 2H), 1.45–1.33 (m, 3H), 1.32–1.17 (overlapping signals: m, 3H and 1.31, s, 3H), 1.08 (d, *J* = 7.4 Hz, 3H), 1.06 (d, *J* = 6.9 Hz, 3H), 1.01–0.95 (m, 1H), 0.91 (s, 3H); ^13^C NMR (126 MHz, MeOD) δ 177.5, 96.2, 79.0, 71.4, 65.8, 52.8, 52.3, 43.2, 41.3, 40.0, 39.5, 36.84, 36.79, 34.7, 34.5, 33.7, 33.2, 31.0, 27.8, 26.4, 21.2, 20.5, 17.4; HRMS(ESI) m/z calcd. for C_25_H_40_O_4_Na [M + Na]^+^: 427.2824, found 427.2824. Crystal data for C_25_H_40_O_4_ (M = 404.57 g/mol): monoclinic, space group *P*2_1_, a = 10.0027(5) Å, b = 9.9958(5) Å, c = 11.4173(4) Å, V = 1112.61(9) Å^3^, Z = 2, T = 120(2) K, μ(CuKα) = 0.627 mm^−1^, Dcalc = 1.208 g/cm^3^, 11,986 reflections measured (5.951° ≤ Θ ≤ 71.714°), 3745 unique (R_int_ = 0.0358), which were used in all calculations. The final R1 was 0.0548 (I > 2σ(I)) and wR2 was 0.1472 (all data).

*Methyl 3α-acetoxy-7α-hydroxyl-12β-methyl-18-nor-5β-chol-13(14)-en-24-oate* (**32**). To a stirred solution of **16** (0.097 g, 0.24 mmol) in THF (15 mL) was added NaHCO_3_ (0.440 g, 5.2 mmol) and acetic anhydride (0.5 mL, 5.0 mmol) and the mixture was heated at 40 °C (18 h). The reaction was quenched with water (30 mL) and extracted with ethyl acetate (30 mL). The organic layer was washed with brine, dried (MgSO_4_), concentrated and the residue was purified by flash chromatography on silica gel (ethyl acetate/petroleum ether, 0:10 to 1:1) to afford the title compound **32** (0.035 g, 33%). ^1^H NMR (500 MHz, CDCl_3_) δ 4.58 (tt, *J* = 11.4, 4.6 Hz, 1H), 4.11 (q, *J* = 2.9 Hz, 1H), 3.64 (s, 3H), 2.65 (d, *J* = 8.6 Hz, 1H), 2.42–2.30 (m, 4H), 2.29–2.10 (m, 4H), 2.01–1.88 (overlapping signals: m, 3H; 1.98, s, 3H), 1.87–1.63 (m, 6H), 1.57–1.40 (m, 3H), 1.29–1.18 (m, 2H), 1.14–0.96 (overlapping signals: m, 2H; 1.04, d, *J* = 7.08 Hz, 3H), 0.93 (d, *J* = 6.95 Hz, 3H), 0.90 (s, 3H); ^13^C NMR (126 MHz, CDCl_3_) δ 174.5, 170.7, 143.1, 137.7, 74.2, 68.2, 53.5, 51.4, 41.6, 40.6, 35.7, 35.3, 35.0, 34.8, 33.9, 33.6, 33.0, 32.7, 31.9, 31.4, 26.7, 25.3, 24.9, 22.3, 21.4, 21.1, 18.9; HRMS(ESI) *m/z* calcd. for C_27_H_42_O_5_Na [M + Na]^+^: 469.2930, found 469.2931.

*Methyl (4R)-4-((1S,3aR,4aR,5aS,7R,9aS,11R,11aR)-7-acetoxy-3a,9a,11-trimethylhexadecahydrobenzo[4,5]indeno[7,1-bc]cyclopenta[d]furan-1-yl)pentanoate* (**33**). Diethylzinc (1 M in hexanes, 1.3 mL, 1.3 mmol) was added to dichloromethane (2.5 mL) and cooled to 0 °C before trifluoroacetic acid (0.1 mL, 1.4 mmol) and diiodomethane (0.4 mL, 5 mmol) were added to the mixture. After 10 min, a solution of **32** (0.057 g, 0.13 mmol) in dichloromethane (3 mL) was added and the reaction mixture was stirred at r.t. (2 h) before being quenched with NH_4_Cl sat. (25 mL). The aqueous layer was washed with ethyl acetate (3 × 20 mL) and the combined organic layers were washed with NaHCO_3_ (20 mL), water (20 mL), brine (25 mL), dried (MgSO_4_) and concentrated. The crude product was purified by flash chromatography on silica gel (ethyl acetate/petroleum ether, 0:10 to 3:7) to afford the title compound **33** (0.030 g, 51% based on a purity of 90%). ^1^H NMR (500 MHz, CDCl_3_) δ 4.71 (tt, *J* = 8.3, 3.9 Hz, 1H), 4.16 (dt, *J* = 5.6, 4.0 Hz, 1H), 3.65 (s, 3H), 2.41 (ddd, *J* = 15.0, 9.4, 5.2 Hz, 1H), 2.31–2.20 (m, 3H), 2.02–1.94 (overlapping signals, m, 1H and 1.98, s, 3H), 1.87–1.74 (m, 6H), 1.70–1.63 (m, 4H), 1.58–1.46 (m, 3H), 1.46–1.34 (m, 3H), 1.29–1.22 (overlapping signals, m, 2H and 1.25, s, 3H), 0.99 (d, *J* = 7.4 Hz, 3H), 0.97 (d, *J* = 6.9 Hz, 3H), 0.94–0.88 (m, 1H), 0.86 (s, 3H); ^13^C NMR (126 MHz, CDCl_3_) δ 174.1, 170.7, 94.4, 72.0, 63.9, 51.5, 51.2, 50.7, 41.9, 40.9, 38.9, 38.5, 35.9, 33.9, 33.8, 33.4, 33.1, 33.0, 32.7, 29.8, 27.3, 26.3, 25.4, 21.5, 20.2, 20.1, 16.8; HRMS(ESI) *m/z* calcd. for C_28_H_44_O_5_Na [M + Na]^+^: 483.3086, found 483.3071.

## Data Availability

CCDC 2150664 (**18**) and 2150665 (**31**) contains the supplementary crystallographic data for this paper. These data can be obtained free of charge from The Cambridge Crystallographic Data Centre via www.ccdc.cam.ac.uk/structures (accessed on 25 February 2022).

## Supplementary Materials

The following supporting information can be downloaded at https://www.mdpi.com/article/10.3390/molecules27072364/s1; spectroscopy for compounds **16**–**25**, **27** and **29**–**33**.

## Sample Availability

Samples of the compounds presented in this research may be made available from the corresponding authors.
