# Supplementary material for: Synthesis of Novel C/D Ring Modified Bile Acids
Source: Molecules. 2022 Apr 6;27(7):2364. doi: 10.3390/molecules27072364 (PMC9000252; doi:10.3390/molecules27072364)

Supplementary Information for:

Synthesis of Novel C/D Ring Modified Bile Acids

Roselis A. Landaeta Aponte [1], Andreas Luxenburger [1], Scott A. Cameron [1], Alex Weymouth-Wilson [2], Richard H. Furneaux [1], Lawrence D. Harris [1,*] and Benjamin J. Compton [1,*]

[1]Ferrier Research Institute, Victoria University of Wellington, Lower Hutt, New Zealand
[2] ICE group, New Zealand Pharmaceuticals Ltd, 68 Weld Street, RD2, Palmerston North 4472, New Zealand
* Correspondence: Lawrence.Harris@vuw.ac.nz; Benji.Compton@vuw.ac.

Contents:

^{1}H NMR spectrum of compound **16** S2
^{13}C NMR spectrum of compound **16** S2
^{1}H NMR spectrum of compound **17** S3
^{13}C NMR spectrum of compound **17** S3
^{1}H NMR spectrum of compound **18** S4
^{13}C NMR spectrum of compound **18** S4
^{1}H NMR spectrum of compound **19** S5
^{13}C NMR spectrum of compound **19** S5
^{1}H NMR spectrum of compound **20** S6
^{13}C NMR spectrum of compound **20** S6
^{1}H NMR spectrum of compound **21** S7
^{13}C NMR spectrum of compound **21** S7
^{1}H NMR spectrum of compound **22** S8
^{13}C NMR spectrum of compound **22** S8
^{1}H NMR spectrum of compound **23** S9
^{13}C NMR spectrum of compound **23** S9
^{1}H NMR spectrum of compound **24** S10
^{13}C NMR spectrum of compound **24** S10
^{1}H NMR spectrum of compound **25** S11
^{13}C NMR spectrum of compound **25** S11
^{1}H NMR spectrum of compound **27** S12
^{13}C NMR spectrum of compound **27** S12
^{1}H NMR spectrum of compound **29** S13
^{13}C NMR spectrum of compound **29** S13
^{1}H NMR spectrum of compound **30** S14
^{13}C NMR spectrum of compound **30** S14
^{1}H NMR spectrum of compound **31** S15
^{13}C NMR spectrum of compound **31** S15
^{1}H NMR spectrum of compound **32** S16
^{13}C NMR spectrum of compound **32** S16
^{1}H NMR spectrum of compound **33** S17
^{13}C NMR spectrum of compound **33** S17

^{1}H NMR spectrum of compound **16** (500 MHz, $CDCl_3$)

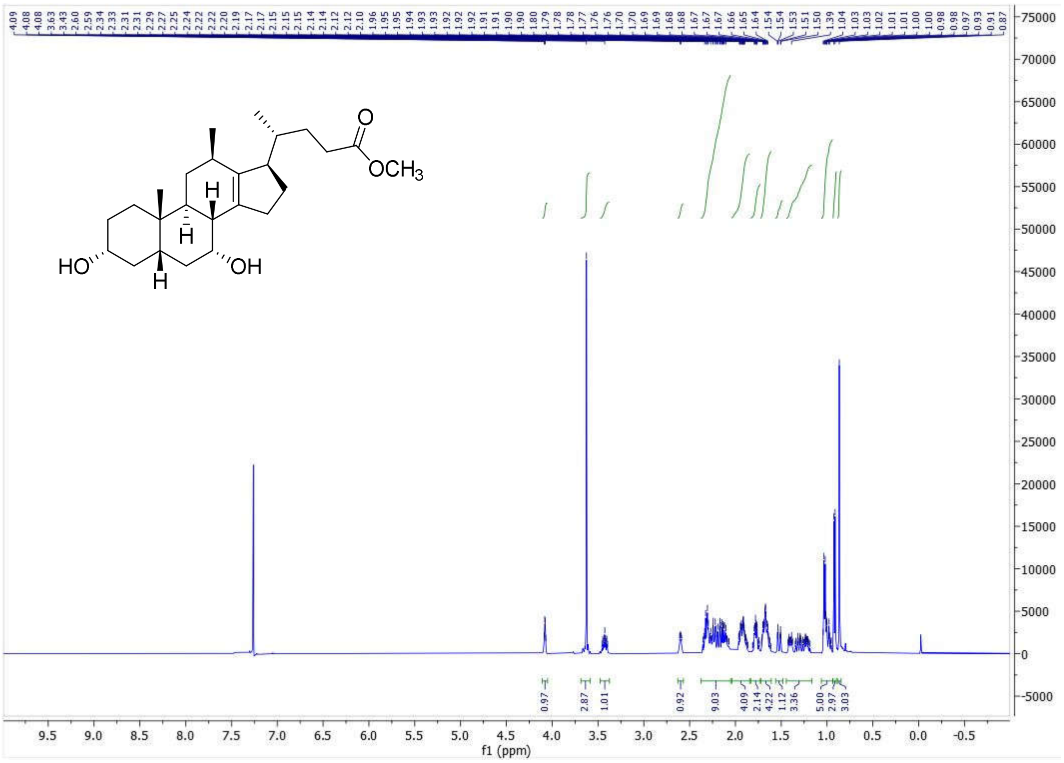

^{13}C NMR spectrum of compound **16** (126 MHz, $CDCl_3$)

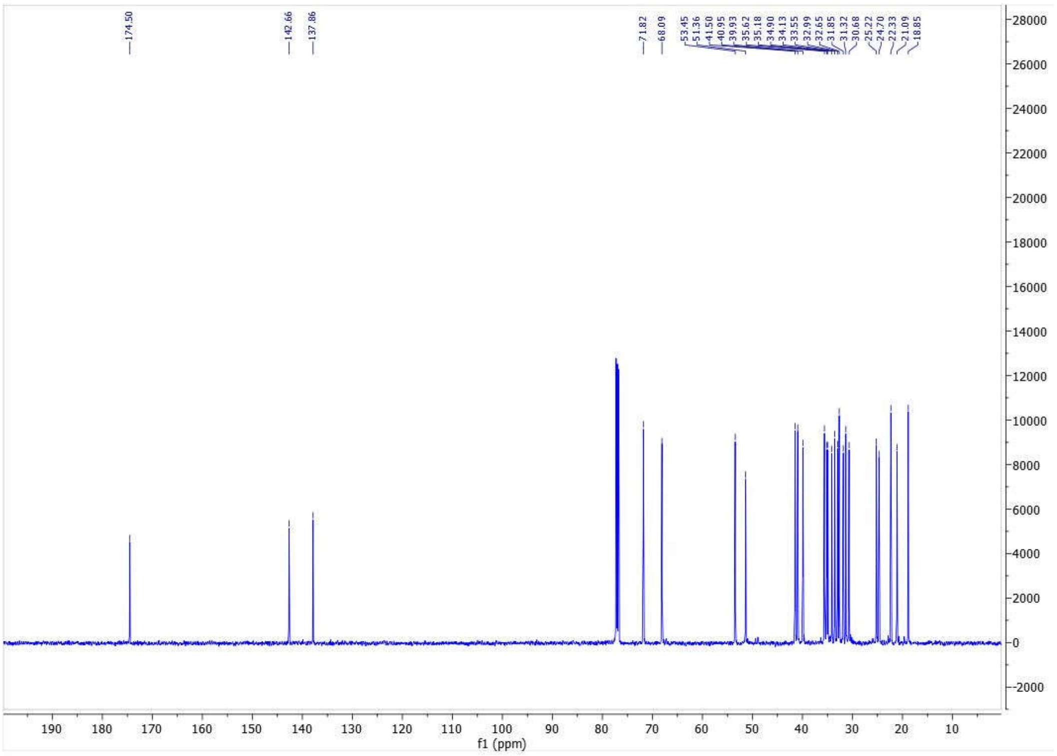

^1H NMR spectrum of compound **17** (500 MHz, $CDCl_3$)

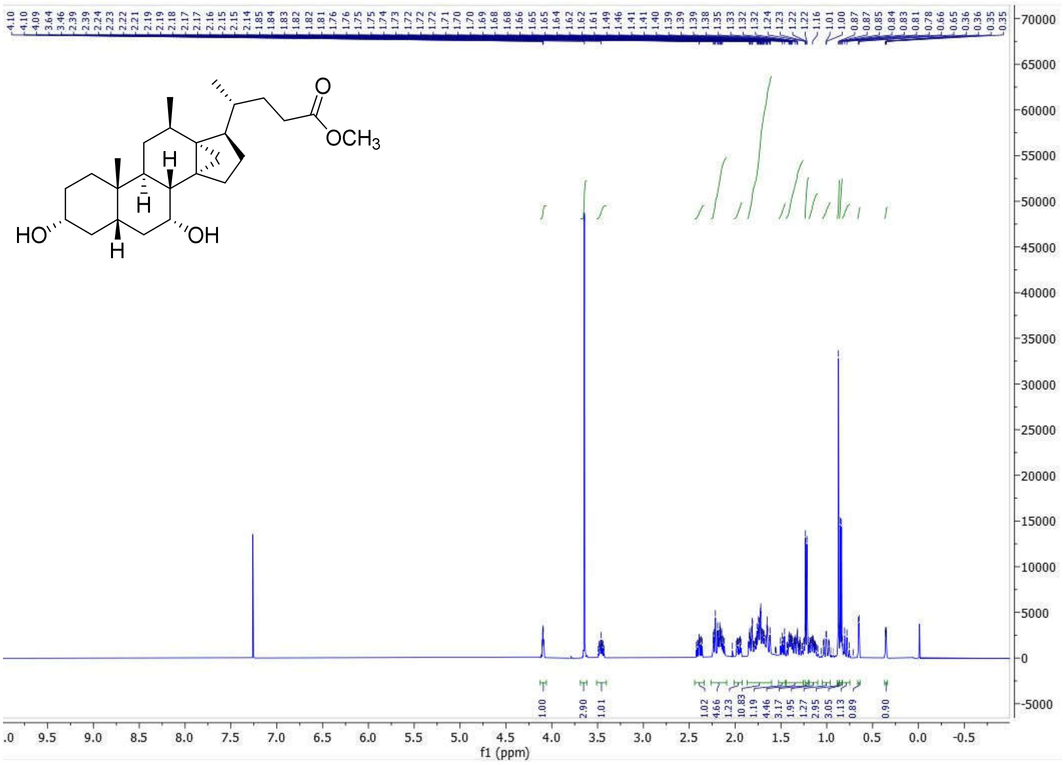

^{13}C NMR spectrum of compound **17** (126 MHz, $CDCl_3$)

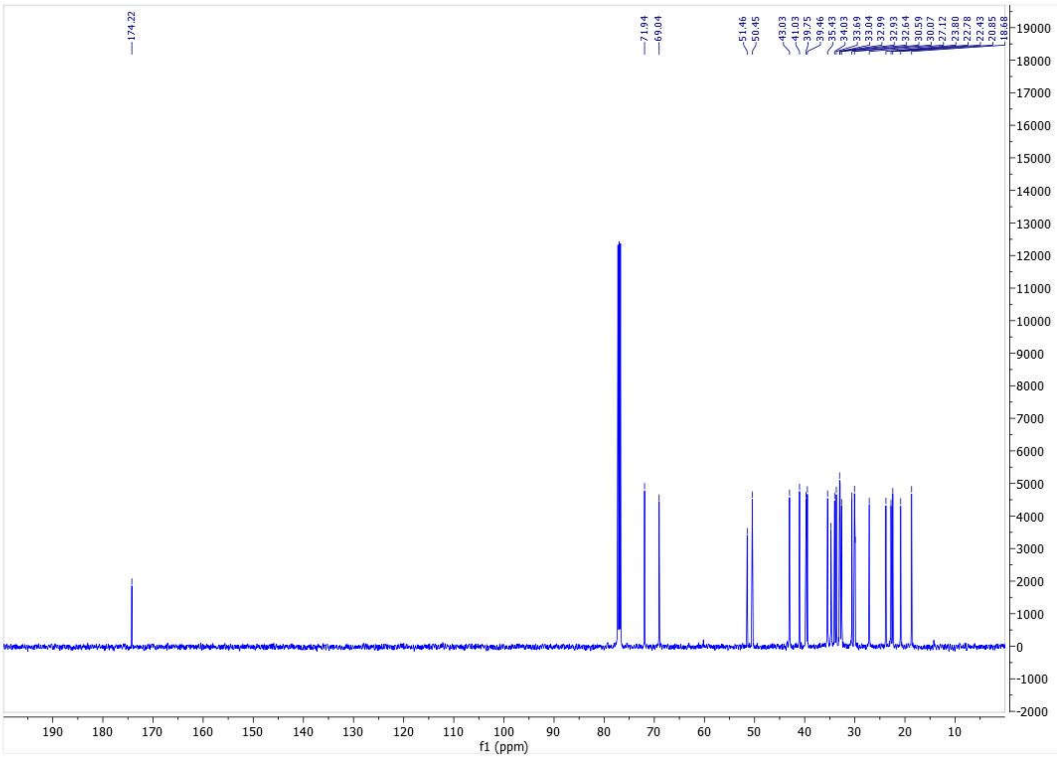

^{1}H NMR spectrum of compound **18** (500 MHz, $CDCl_3$)

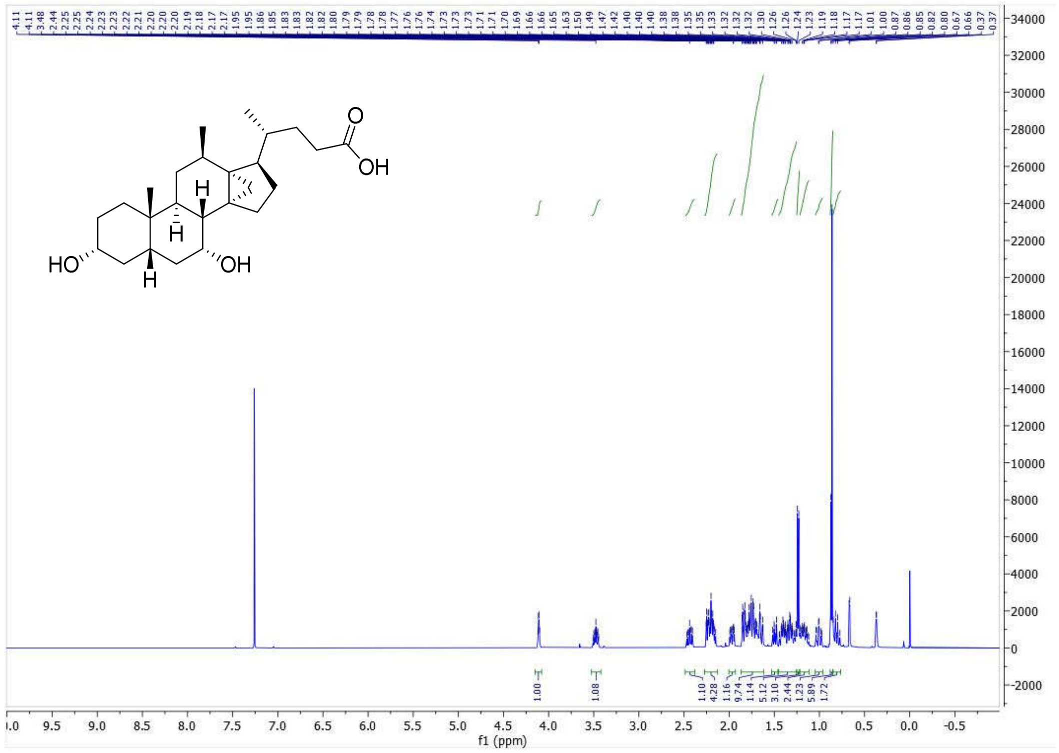

^{13}C NMR spectrum of compound **18** (126 MHz, $CDCl_3$)

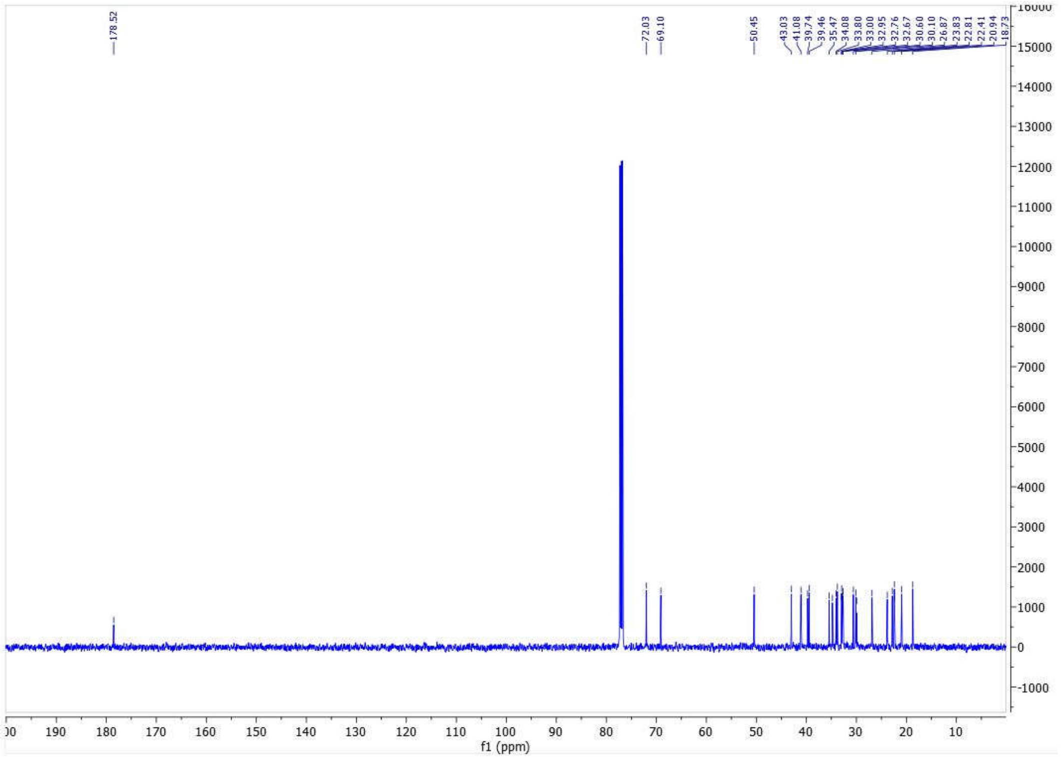

^{1}H NMR spectrum of compound **19** (500 MHz, CD_3OD)

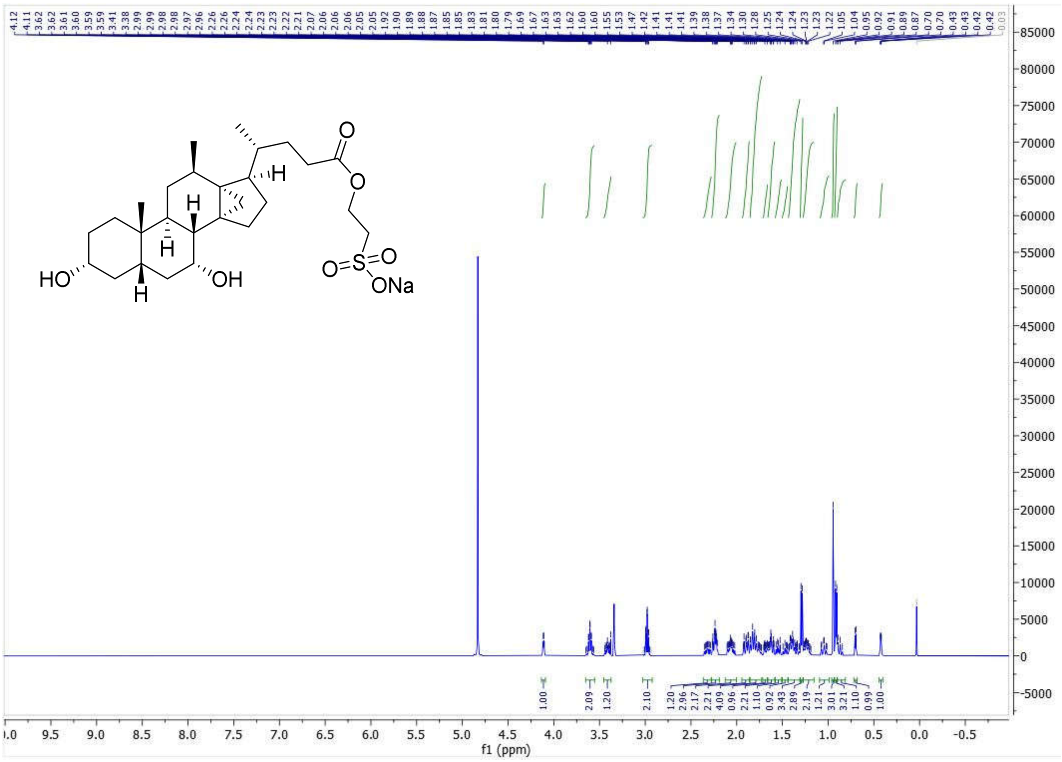

^{13}C NMR spectrum of compound **19** (126 MHz, CD_3OD)

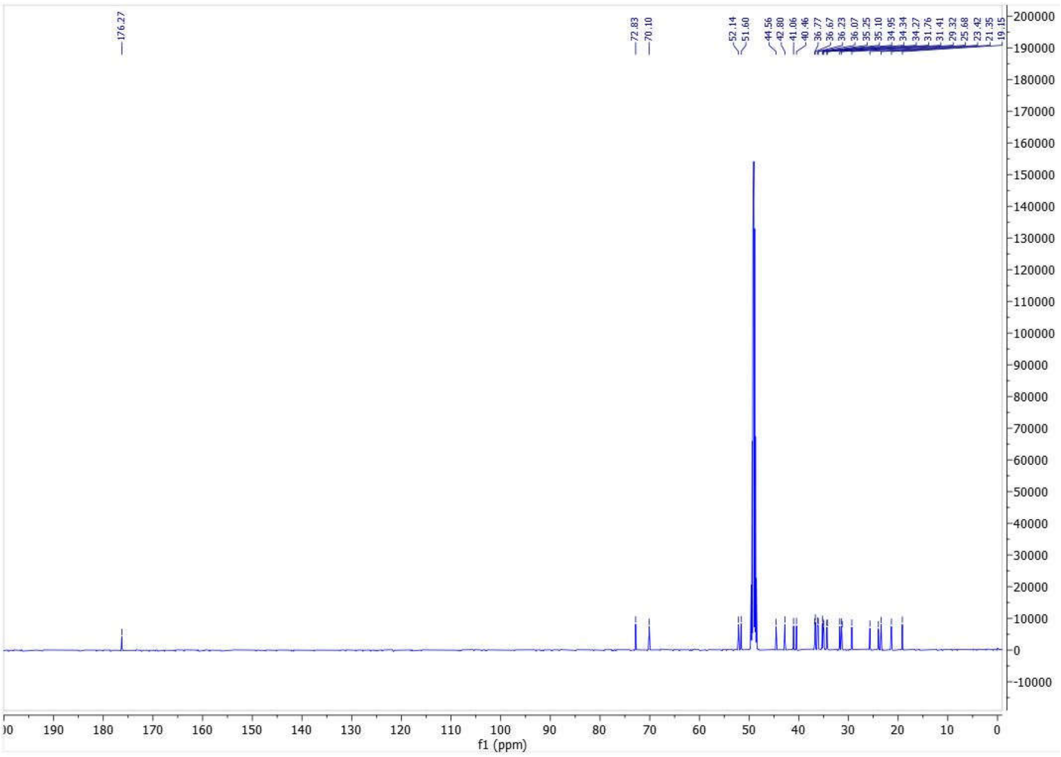

^{1}H NMR spectrum of compound **20** (500 MHz, $CDCl_3$)

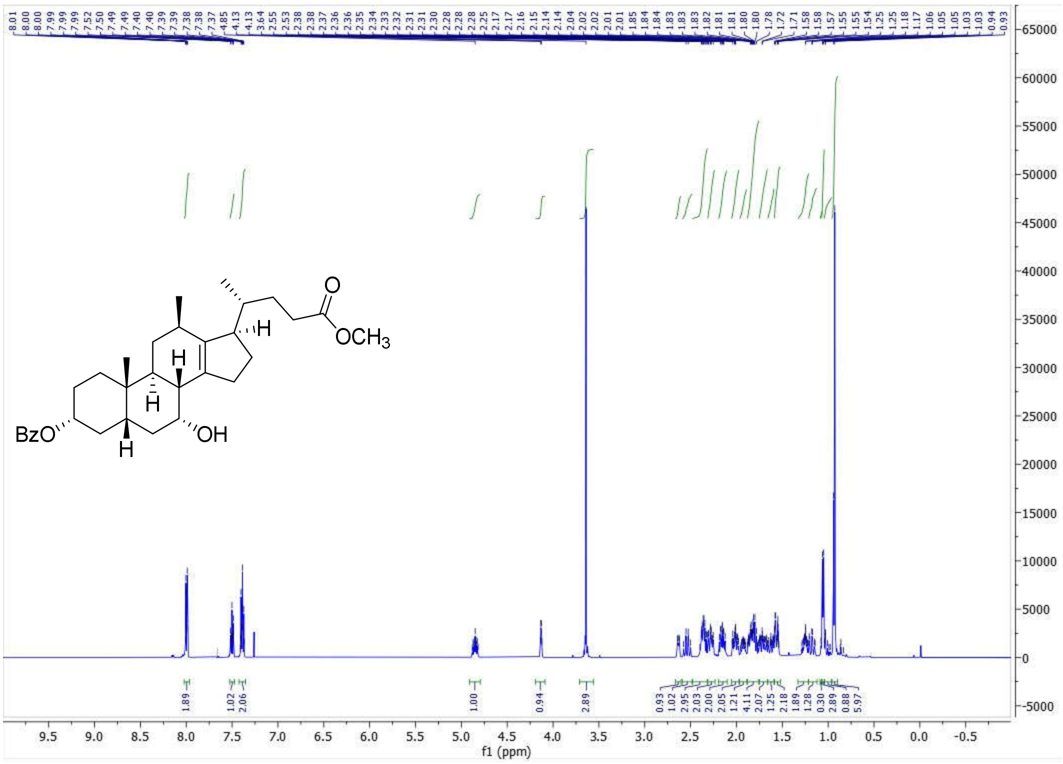

^{13}C NMR spectrum of compound **20** (126 MHz, $CDCl_3$)

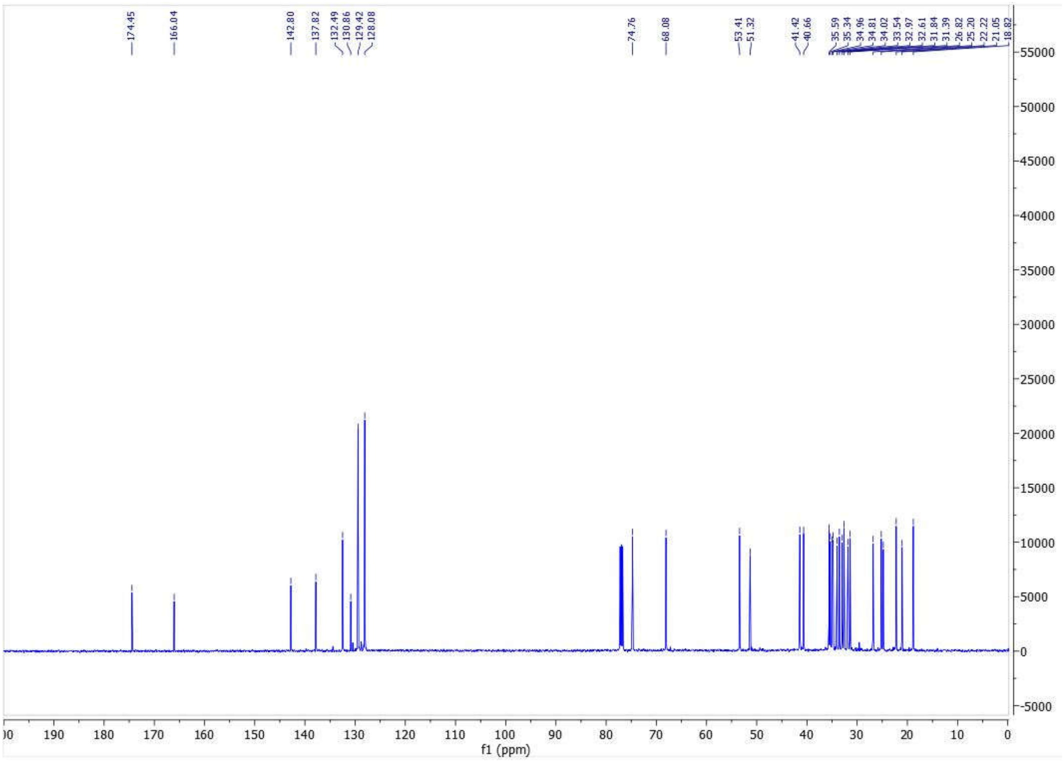

^{1}H NMR spectrum of compound **21** (500 MHz, $CDCl_3$)

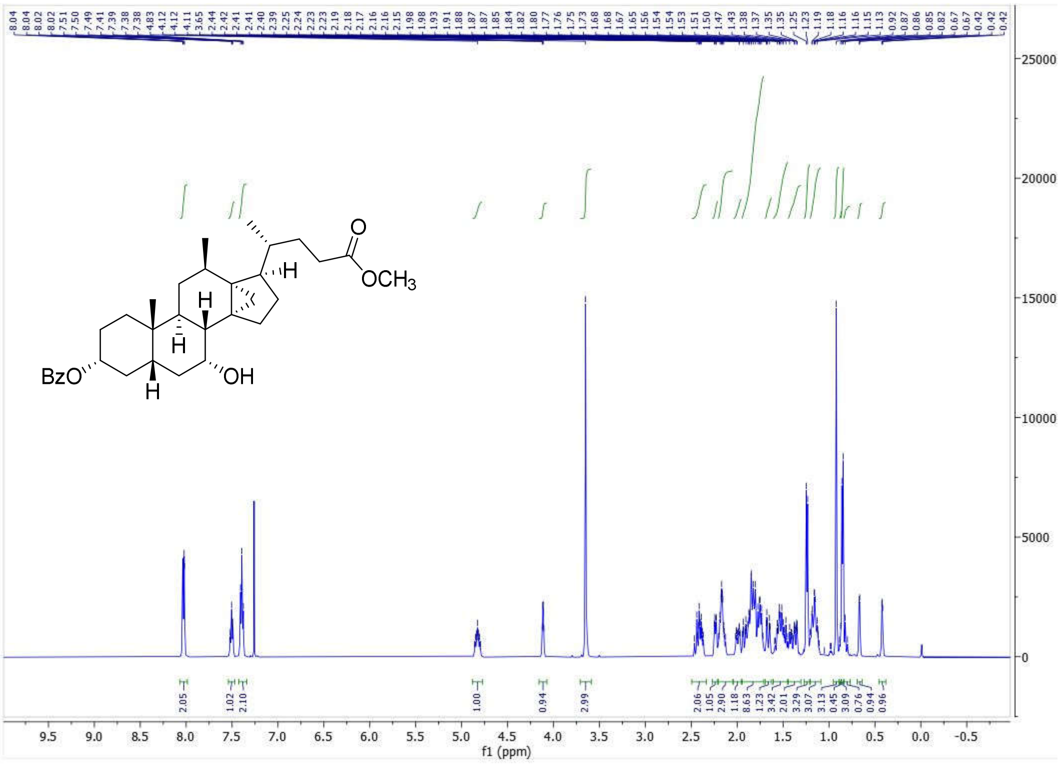

^{13}C NMR spectrum of compound **21** (126 MHz, $CDCl_3$)

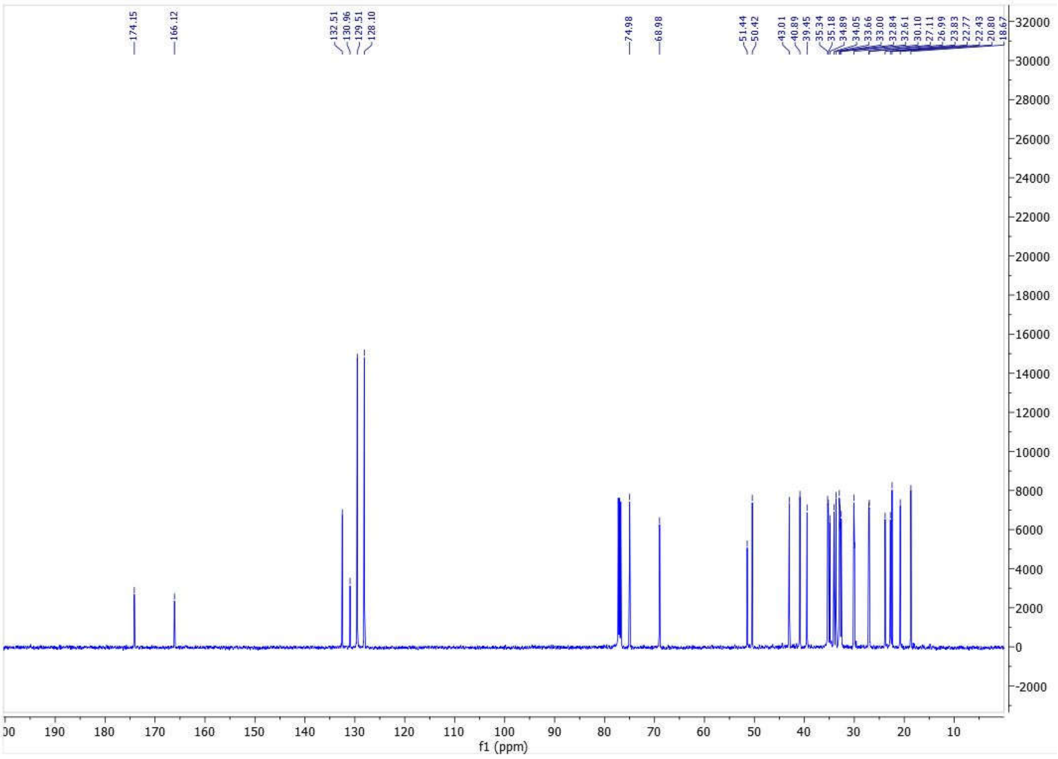

^1H NMR spectrum of compound **22** (500 MHz, $CDCl_3$)

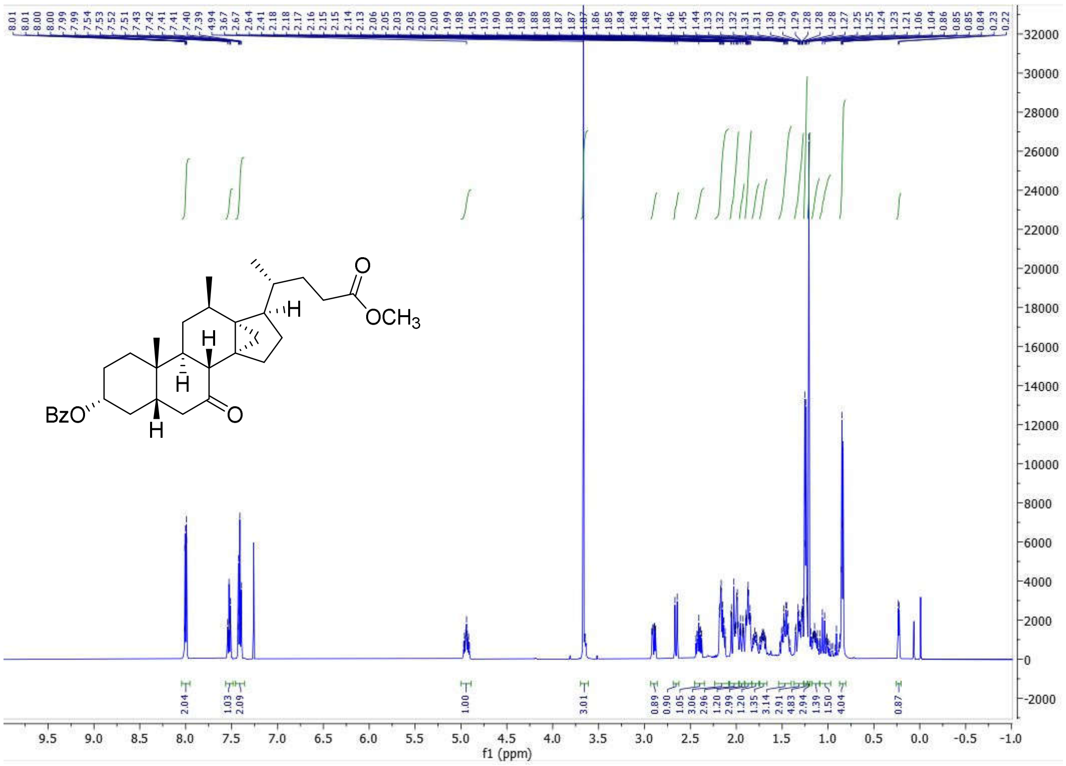

^{13}C NMR spectrum of compound **22** (126 MHz, $CDCl_3$)

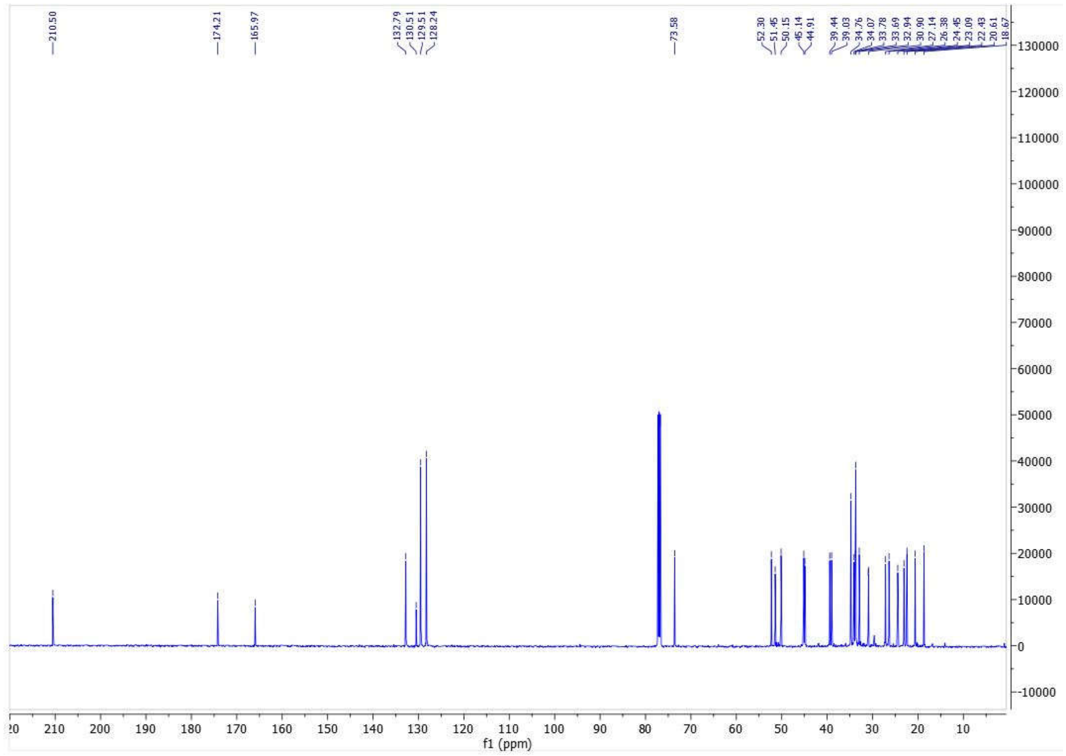

^{1}H NMR spectrum of compound **23** (500 MHz, CD_3OD)

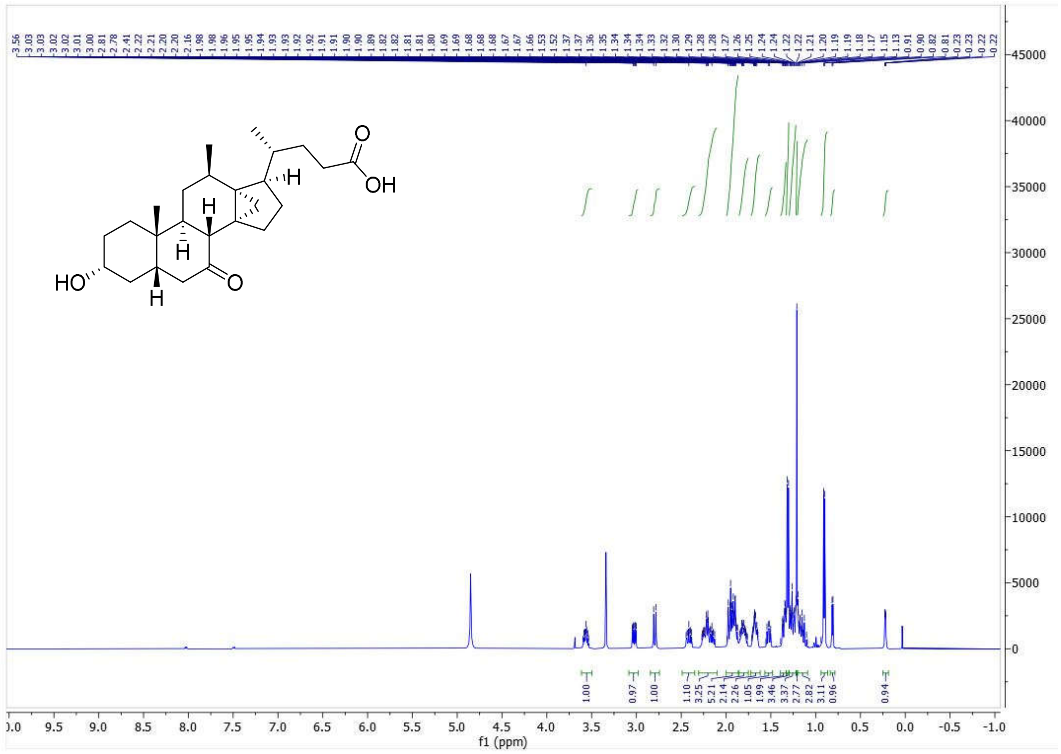

^{13}C NMR spectrum of compound **23** (126 MHz, CD_3OD)

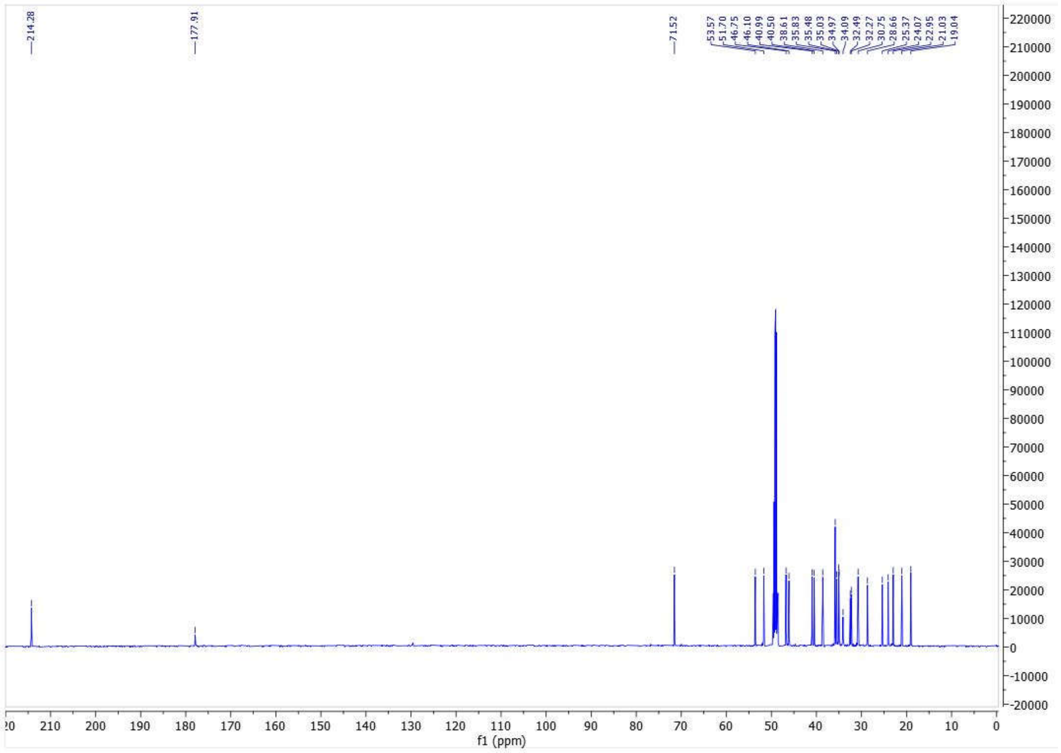

^{1}H NMR spectrum of compound **24** (500 MHz, CD_3OD)

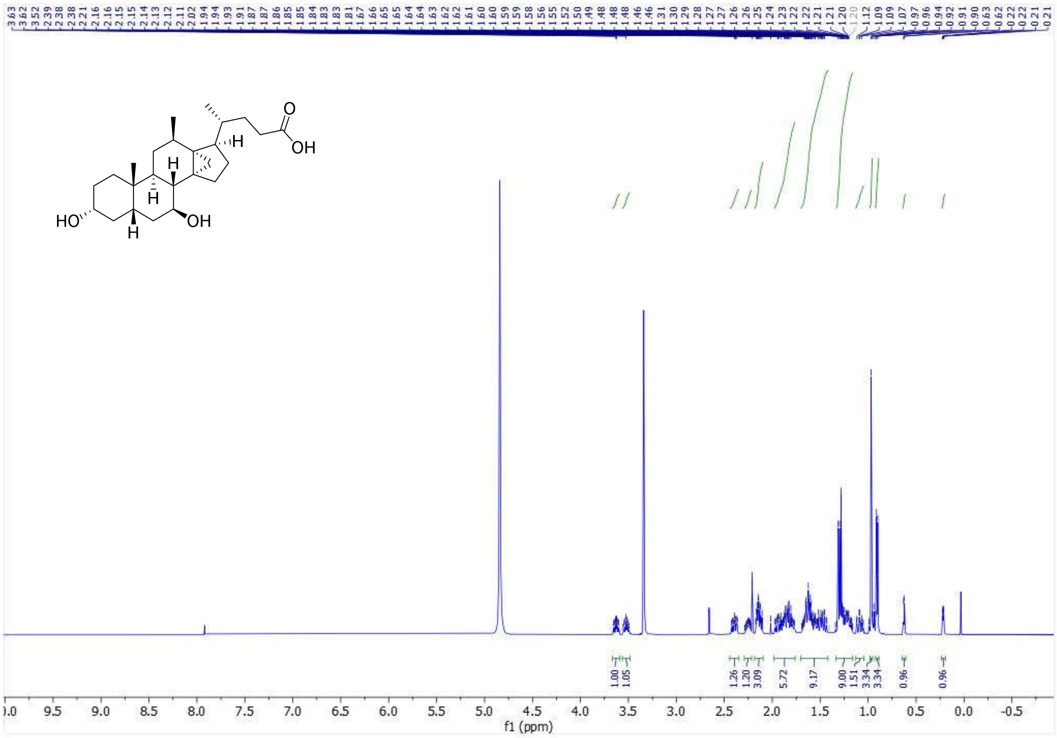

^{13}C NMR spectrum of compound **24** (126 MHz, CD_3OD)

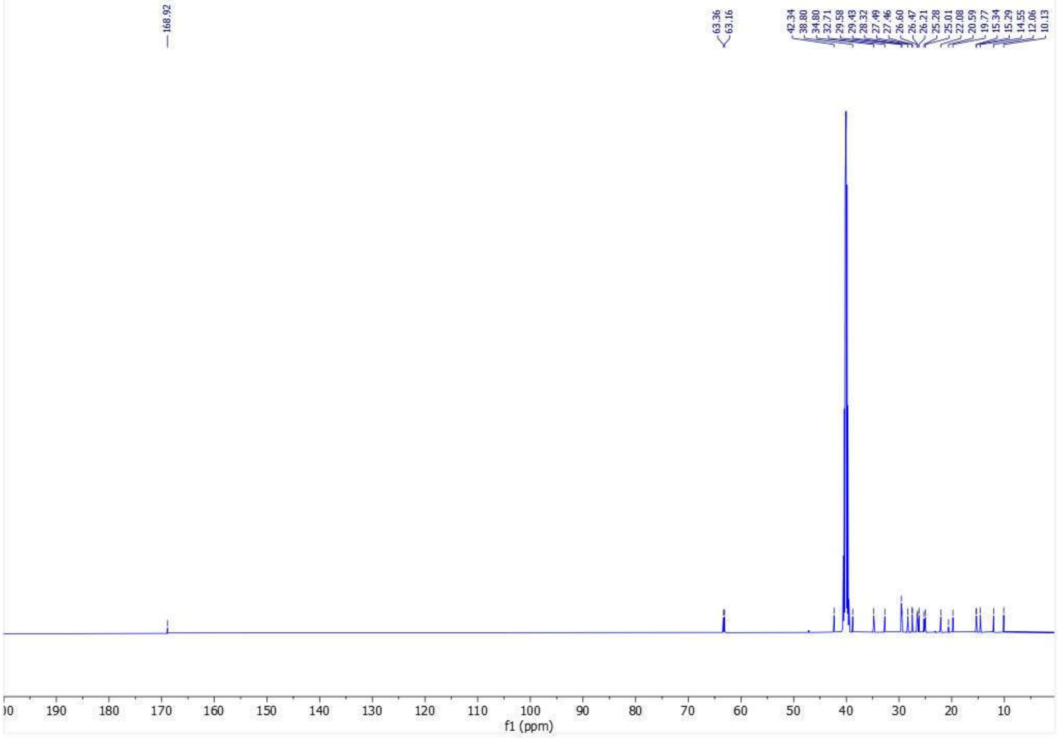

^{1}H NMR spectrum of compound **25** (500 MHz, $CDCl_3$)

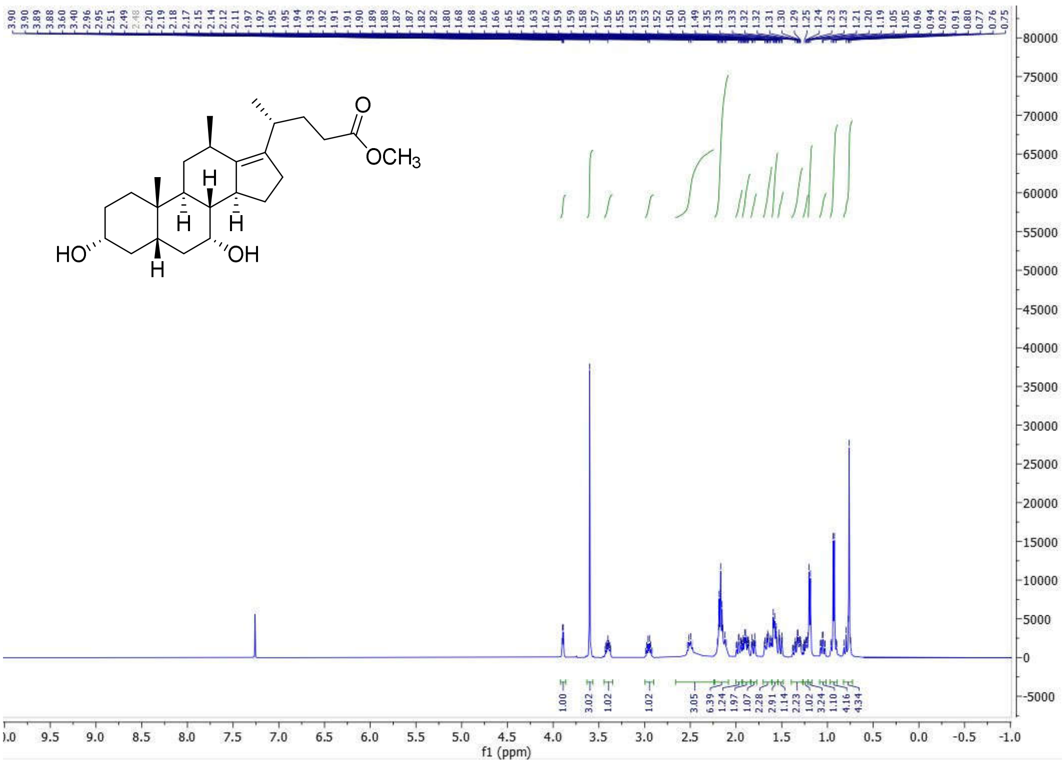

^{13}C NMR spectrum of compound **25** (126 MHz, $CDCl_3$)

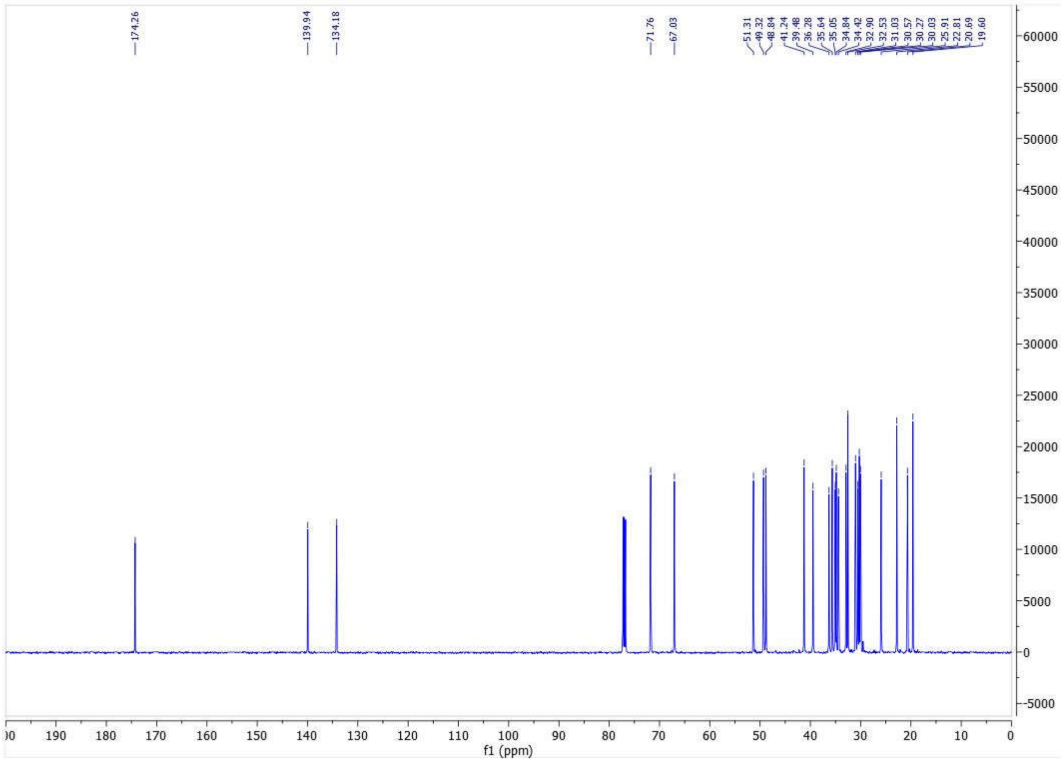

^{1}H NMR spectrum of compound **27** (500 MHz, $CDCl_3$)

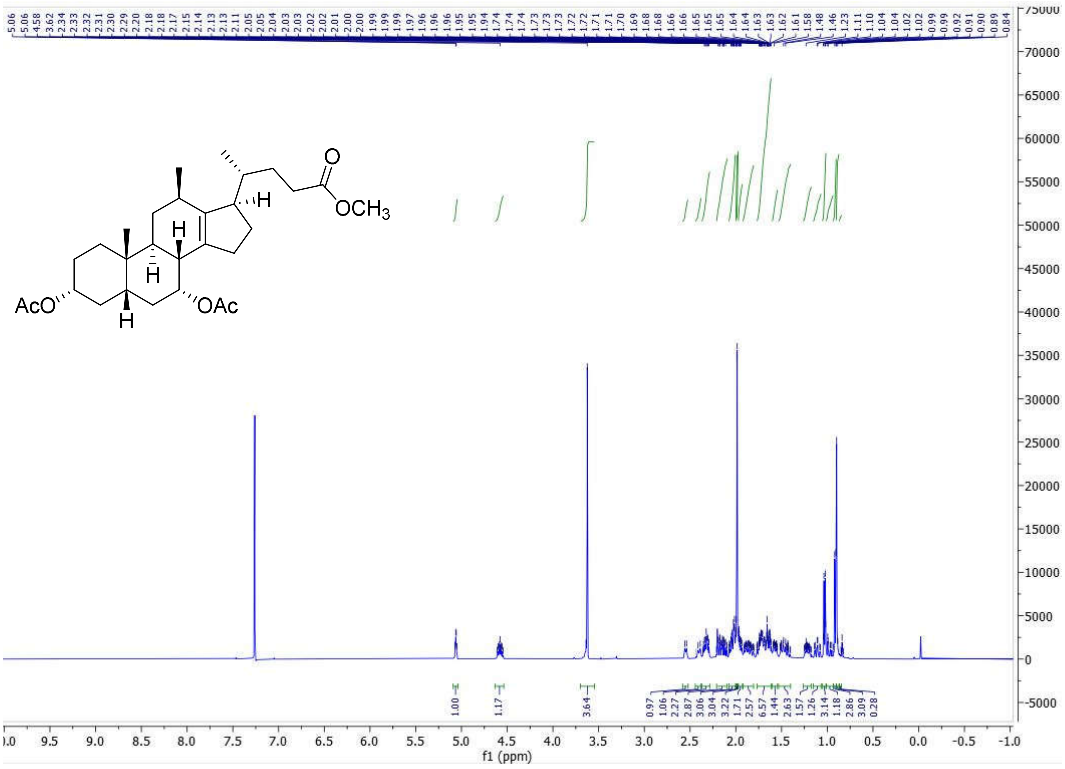

^{13}C NMR spectrum of compound **27** (126 MHz, $CDCl_3$)

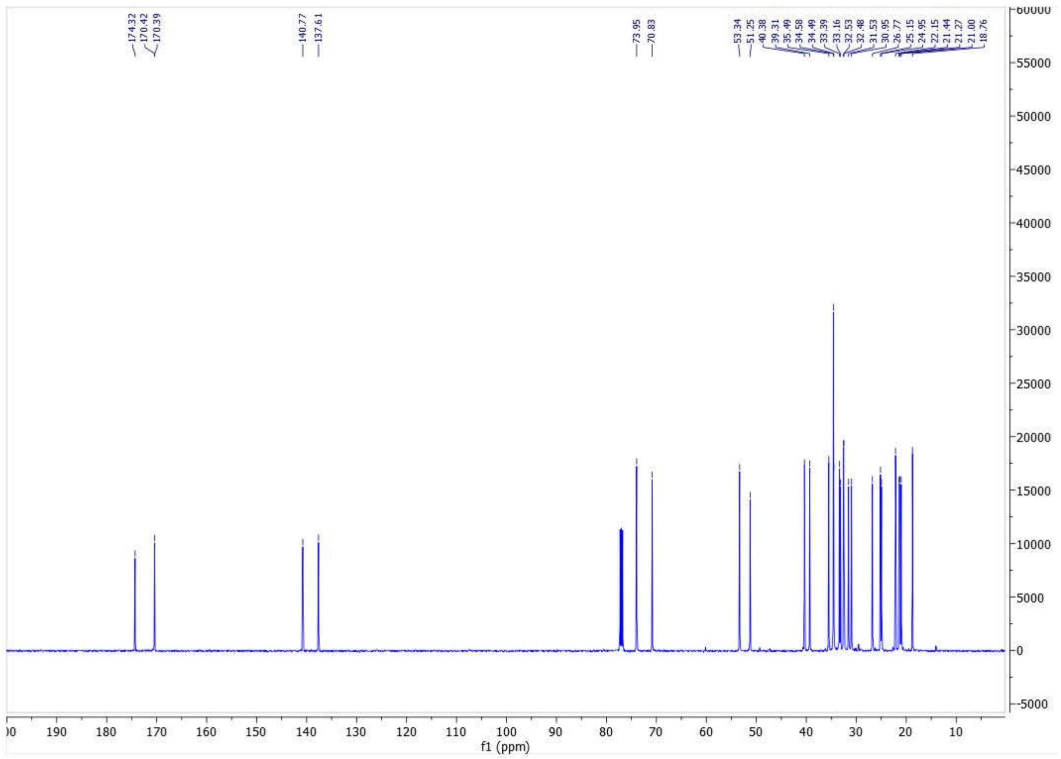

^{1}H NMR spectrum of compound **29** (500 MHz, $CDCl_3$)

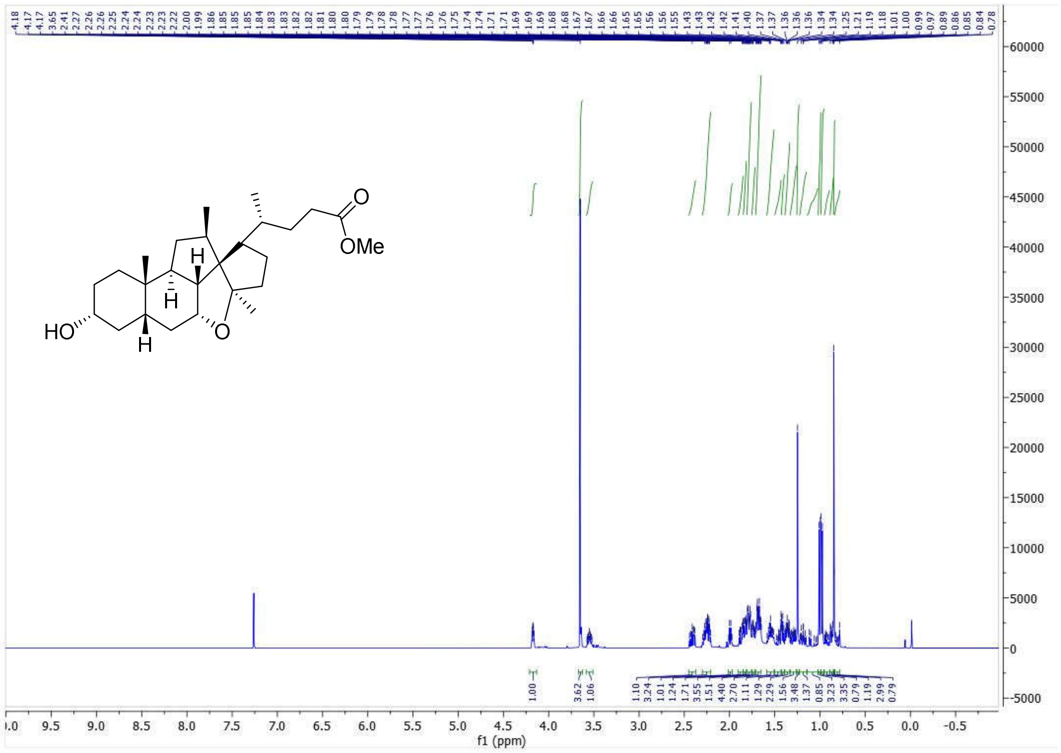

^{13}C NMR spectrum of compound **29** (126 MHz, $CDCl_3$)

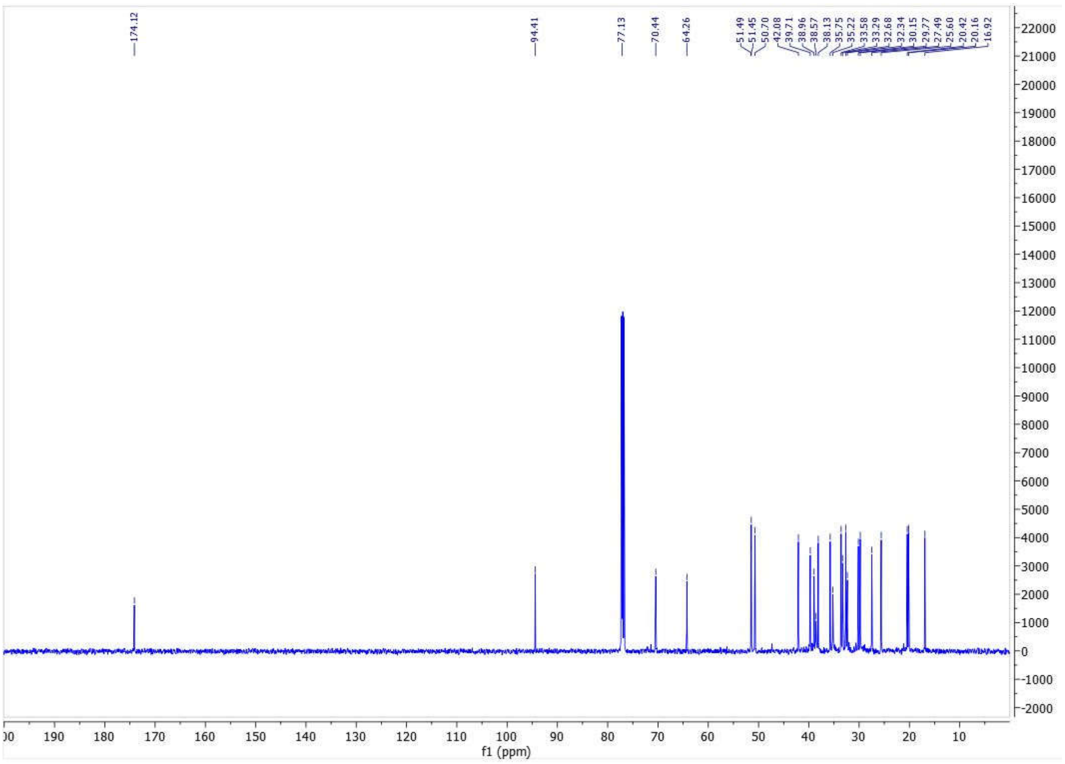

^{1}H NMR spectrum of compound **30** (500 MHz, $CDCl_3$)

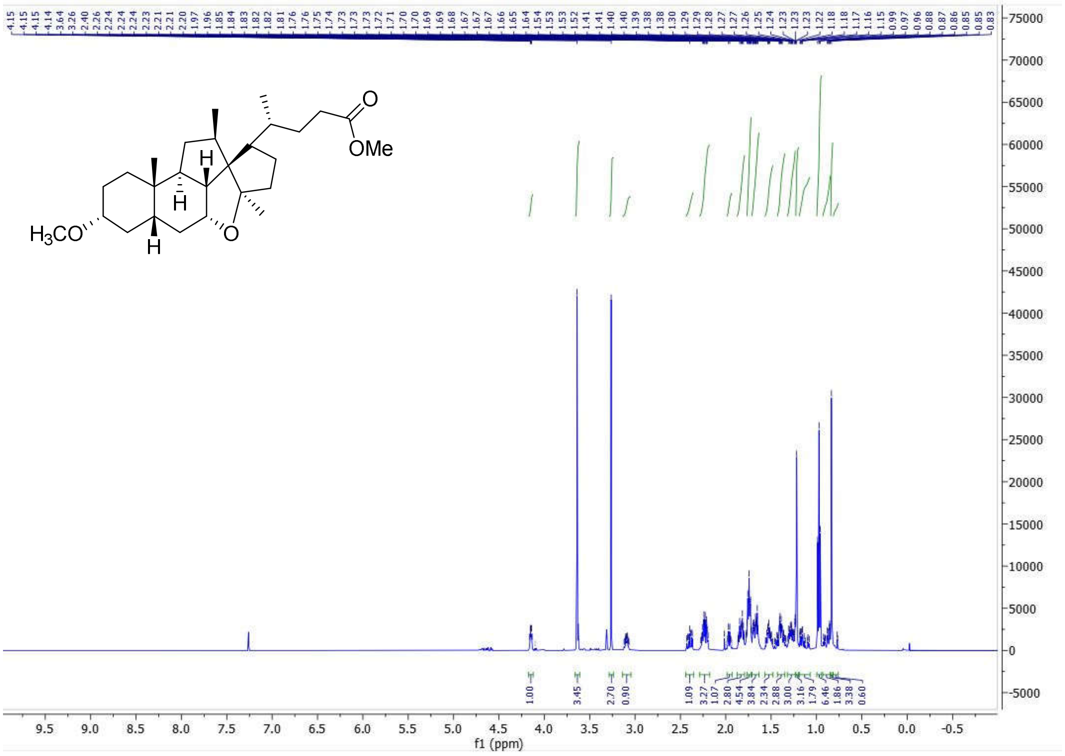

^{13}C NMR spectrum of compound **30** (126 MHz, $CDCl_3$)

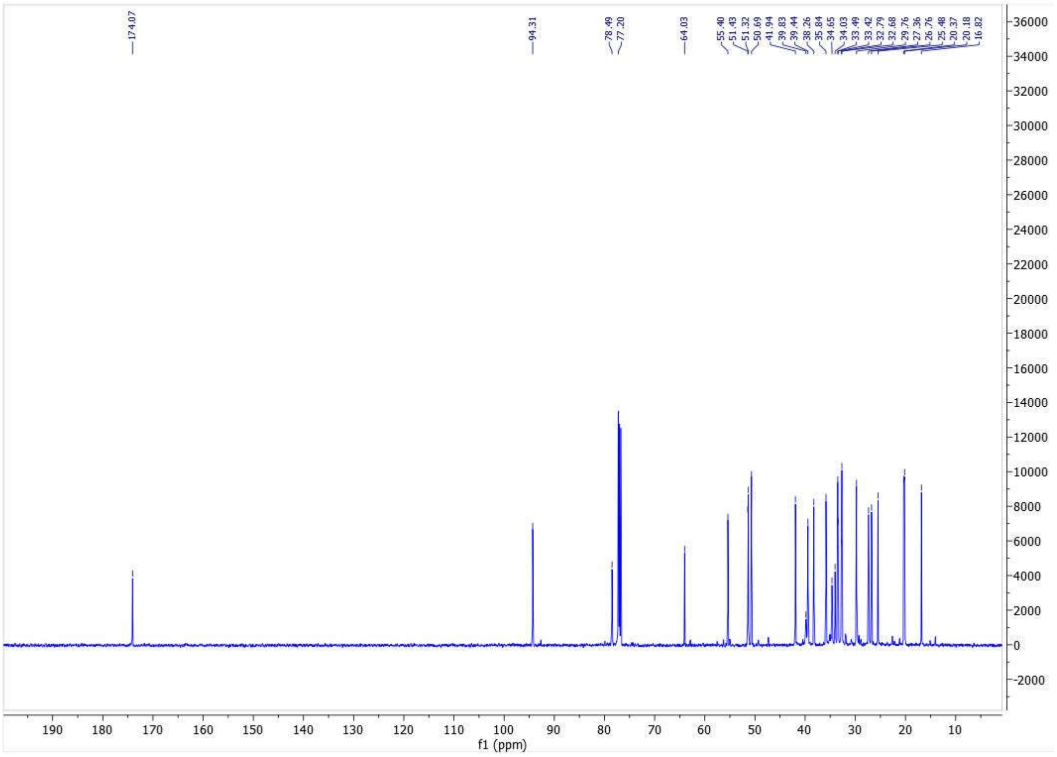

^{1}H NMR spectrum of compound **31** (500 MHz, CD_3OD)

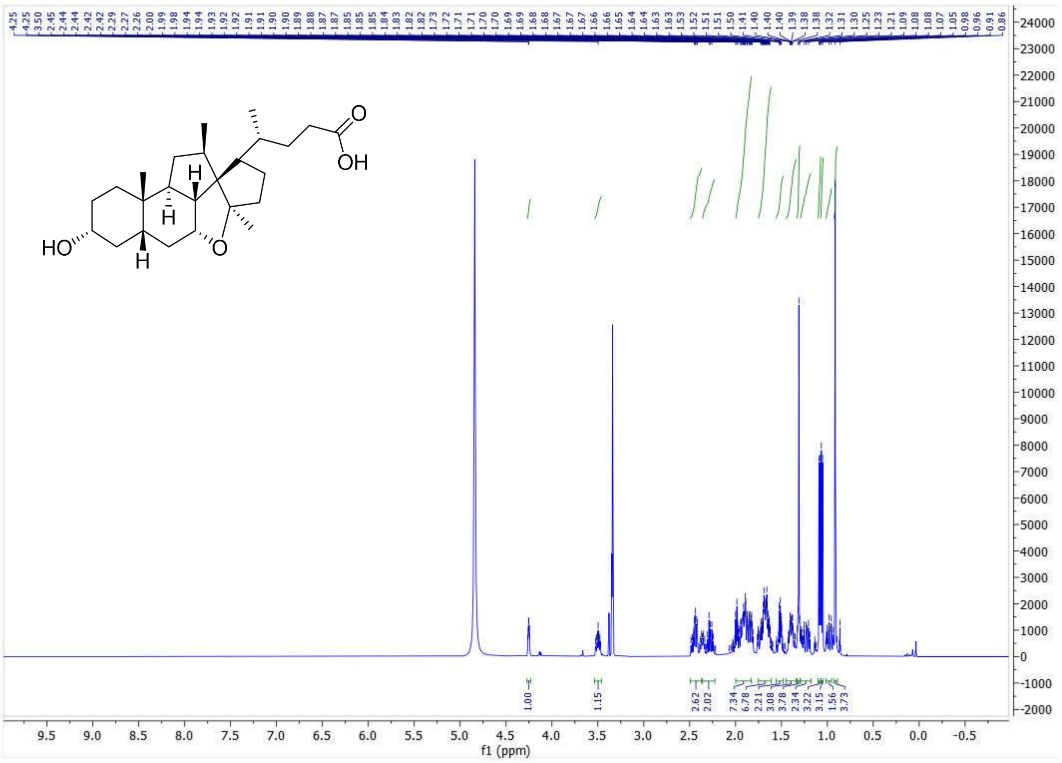

^{13}C NMR spectrum of compound **31** (126 MHz, CD_3OD)

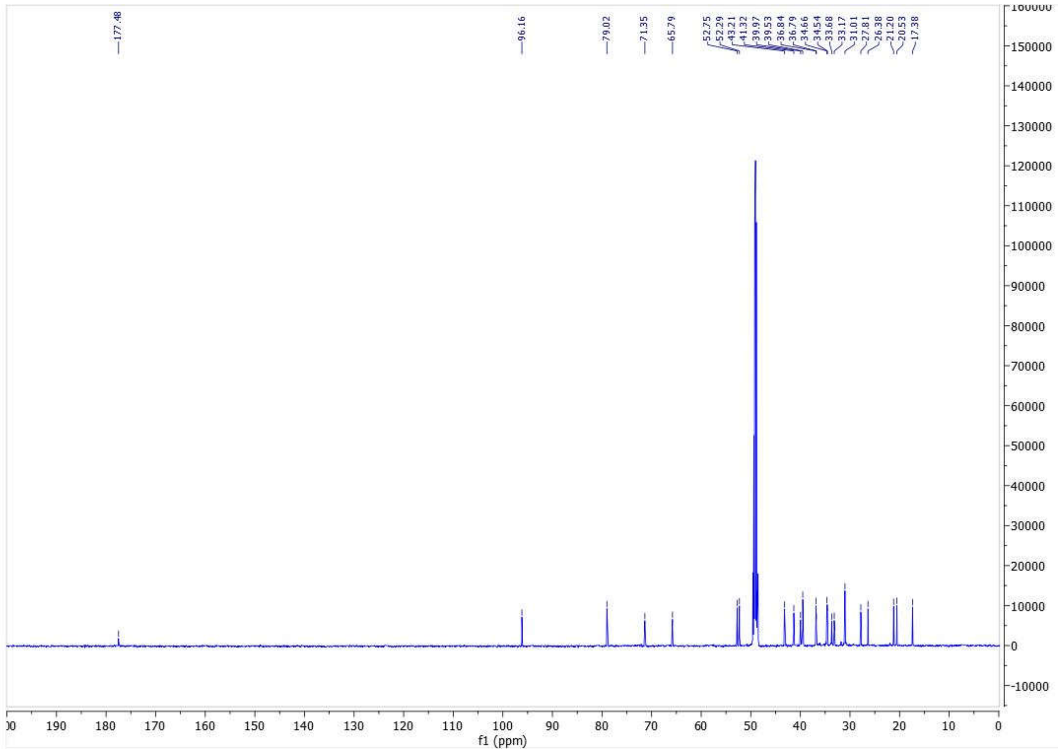

^{1}H NMR spectrum of compound **32** (500 MHz, $CDCl_3$)

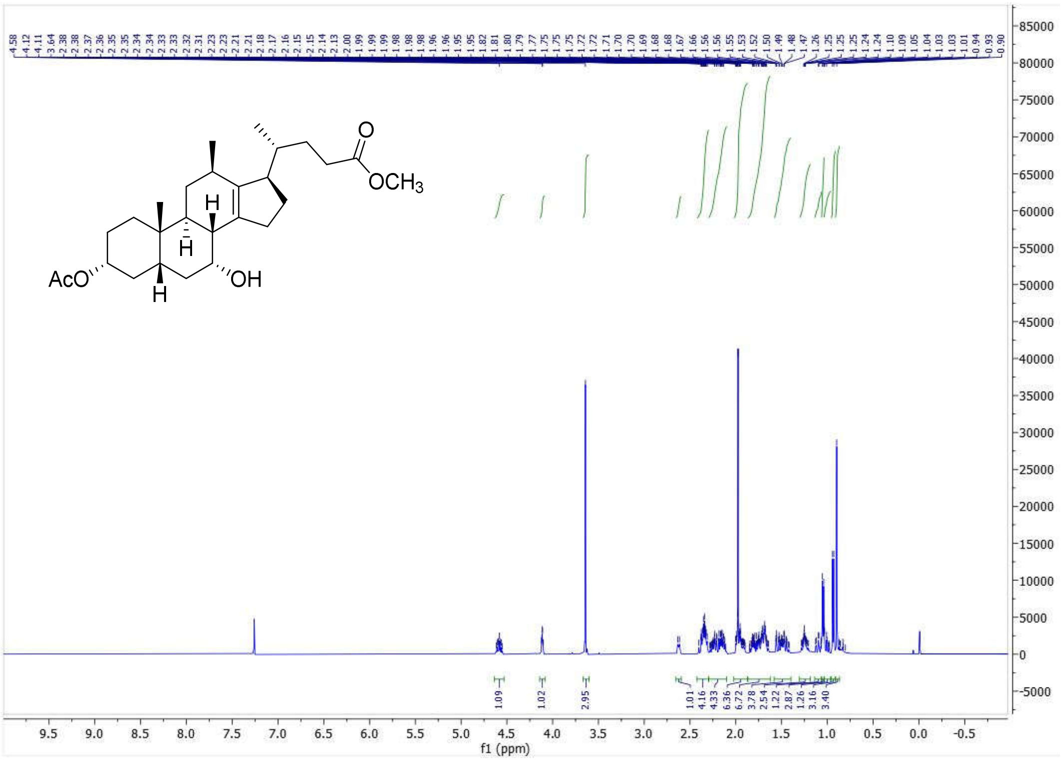

^{13}C NMR spectrum of compound **32** (126 MHz, $CDCl_3$)

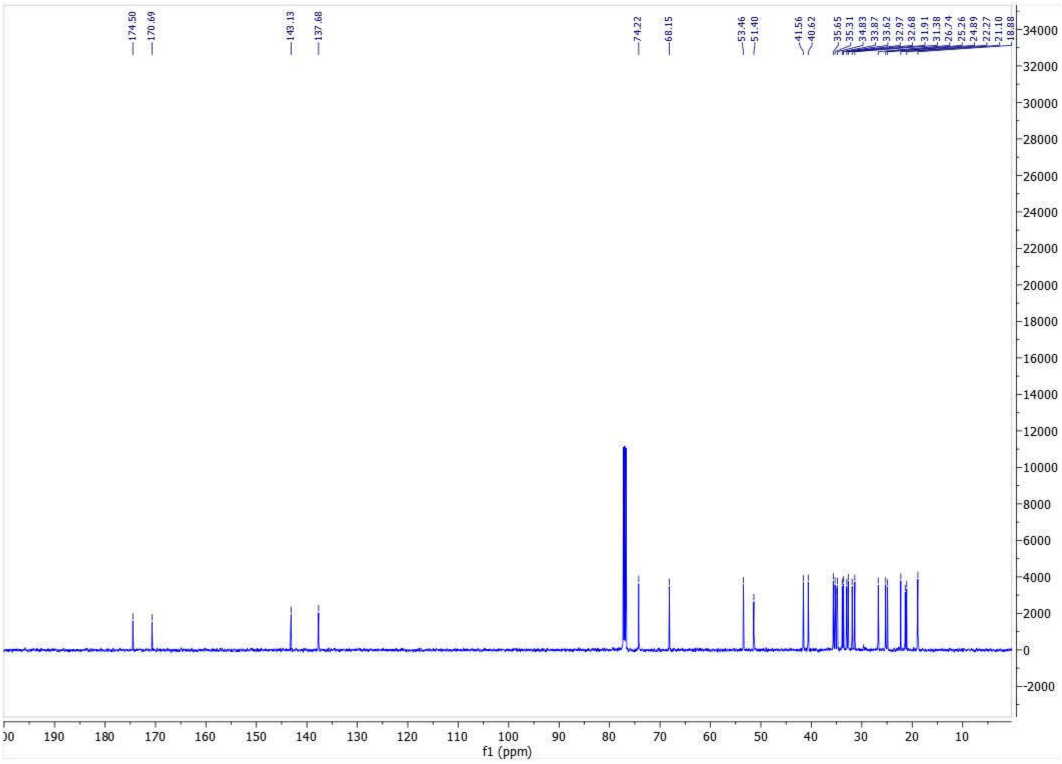

^{1}H NMR spectrum of compound **33** (500 MHz, $CDCl_3$)

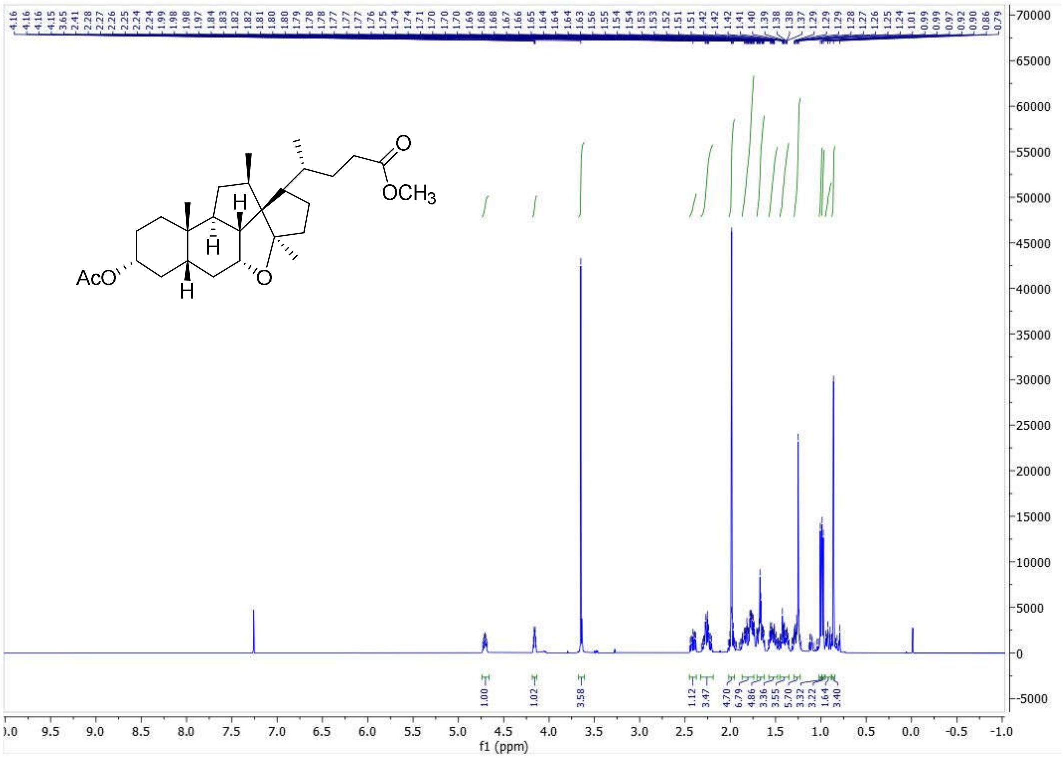

^{13}C NMR spectrum of compound **33** (126 MHz, $CDCl_3$)

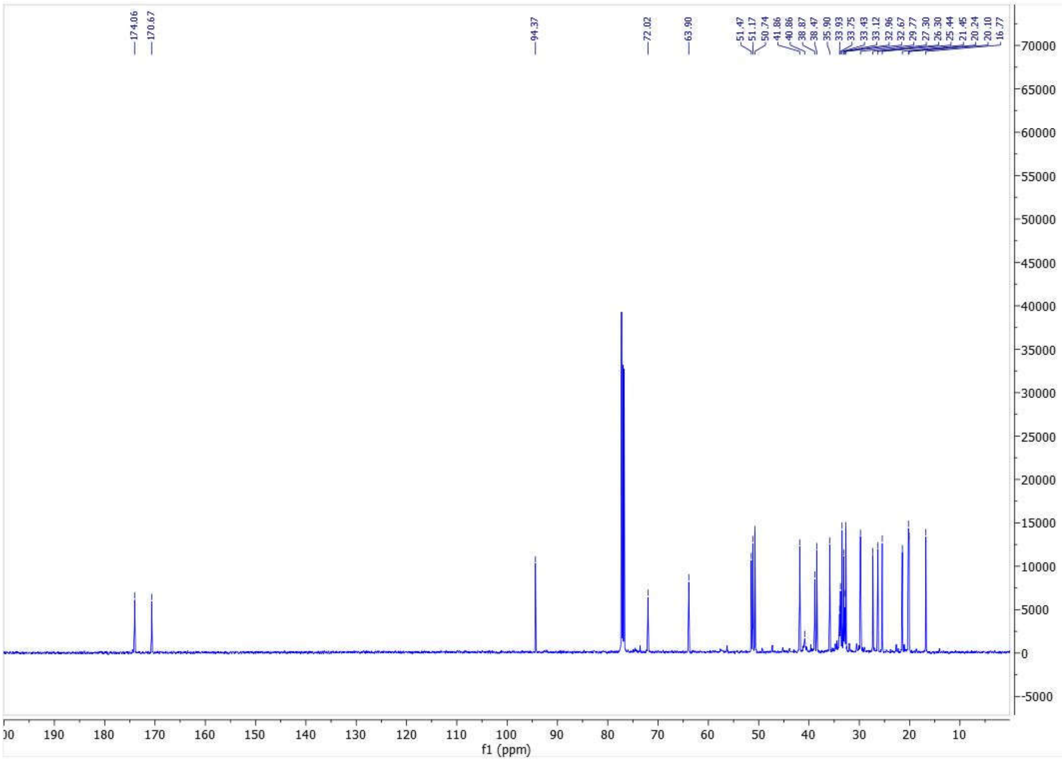

Supplement: Supplementary file 1 [file molecules-27-02364-s001.zip › molecules-1635277-supplementary.pdf]
